# The Parkinson's disease VPS35[D620N] mutation enhances LRRK2-mediated Rab protein phosphorylation in mouse and human

**DOI:** 10.1042/BCJ20180248

**Published:** 2018-06-06

**Authors:** Rafeeq Mir, Francesca Tonelli, Pawel Lis, Thomas Macartney, Nicole K. Polinski, Terina N. Martinez, Meng-Yun Chou, Andrew J.M. Howden, Theresa König, Christoph Hotzy, Ivan Milenkovic, Thomas Brücke, Alexander Zimprich, Esther Sammler, Dario R. Alessi

**Affiliations:** 1 MRC Protein Phosphorylation and Ubiquitylation Unit, School of Life Sciences, University of Dundee, Dundee DD1 5EH, U.K.; 2 The Michael J. Fox Foundation for Parkinson's Research, Grand Central Station, PO Box 4777, New York, NY 10163, U.S.A.; 3 Abcam, 863 Mitten Rd, Burlingame, CA 94010, U.S.A.; 4 Division of Cell Signalling and Immunology, School of Life Sciences, University of Dundee, Dundee, U.K.; 5 Department of Neurology, Medical University of Vienna, Währinger Gürtel 18-20, 1090 Wien, Austria; 6 Verein zur Förderung der wissenschaftlichen Forschung im, Wilhelminenspital (FWFW), Montleartstrasse 37, 1160 Wien, Austria; 7 Department of Neurology, School of Medicine, Dundee, Ninewells Hospital, Ninewells Drive, Dundee DD1 9SY, U.K.

**Keywords:** kinase, Leucine-rich repeat kinase, Parkinson's disease signalling, phosphorylation, Rab proteins, VPS35

## Abstract

Missense mutations in the LRRK2 (Leucine-rich repeat protein kinase-2) and VPS35 genes result in autosomal dominant Parkinson's disease. The VPS35 gene encodes for the cargo-binding component of the retromer complex, while LRRK2 modulates vesicular trafficking by phosphorylating a subgroup of Rab proteins. Pathogenic mutations in LRRK2 increase its kinase activity. It is not known how the only thus far described pathogenic VPS35 mutation, [p.D620N] exerts its effects. We reveal that the VPS35[D620N] knock-in mutation strikingly elevates LRRK2-mediated phosphorylation of Rab8A, Rab10, and Rab12 in mouse embryonic fibroblasts. The VPS35[D620N] mutation also increases Rab10 phosphorylation in mouse tissues (the lung, kidney, spleen, and brain). Furthermore, LRRK2-mediated Rab10 phosphorylation is increased in neutrophils as well as monocytes isolated from three Parkinson's patients with a heterozygous VPS35[D620N] mutation compared with healthy donors and idiopathic Parkinson's patients. LRRK2-mediated Rab10 phosphorylation is significantly suppressed by knock-out or knock-down of VPS35 in wild-type, LRRK2[R1441C], or VPS35[D620N] cells. Finally, VPS35[D620N] mutation promotes Rab10 phosphorylation more potently than LRRK2 pathogenic mutations. Available data suggest that Parkinson's patients with VPS35[D620N] develop the disease at a younger age than those with LRRK2 mutations. Our observations indicate that VPS35 controls LRRK2 activity and that the VPS35[D620N] mutation results in a gain of function, potentially causing PD through hyperactivation of the LRRK2 kinase. Our findings suggest that it may be possible to elaborate compounds that target the retromer complex to suppress LRRK2 activity. Moreover, patients with VPS35[D620N] associated Parkinson's might benefit from LRRK2 inhibitor treatment that have entered clinical trials in humans.

## Introduction

Parkinson's disease (PD) is a common neurodegenerative condition with numbers expected to rise [1]. There is currently no test for diagnosis, cure, or disease-modifying therapies available. Although most PD cases are idiopathic, studying the disease mechanisms of the rarer genetic PD forms is expected to advance our knowledge in the aetiology of idiopathic disease and help identify targets for the development of biomarker and novel treatments. Mutations in at least 18 genes have been linked to heritable forms of PD and may be indistinguishable from idiopathic disease [2,3]. That mutations in LRRK2 (Leucine-rich repeat protein kinase-2) result in PD was first described in 2004 [4,5] and are one of the most common genetic causes for PD. For example, the most frequent LRRK2 mutation (G2019S) is found in 4% of familial and up to 1–2% of sporadic PD patients worldwide [3,6] and with much higher frequency, for example, in Ashkenazi Jews (28% of familial and 10% of sporadic PD) and North African Berbers (40% of familial and 30% of sporadic cases) [4,5,7]. LRRK2 is a large multi-domain protein kinase consisting of a ROC-type GTPase domain in addition to a serine/threonine protein kinase domain [8]. The best characterised pathogenic mutations lie within the ROC domain (R1441C/G, Y1699C) and the kinase domain (G2019S) [9]. The G2019S mutation directly increases the kinase activity of recombinant LRRK2 [9], while the R1441G/C and Y1699C mutations do so indirectly by promoting the recruitment of LRRK2 to the Rab29 protein located at the Golgi, leading to LRRK2 activation through an as yet unknown mechanism [10–12].

Recent work has identified a subgroup of up to 14 endogenous Rab GTPase proteins (Rab3A/B/C/D, Rab5A/B/C, Rab8A/B, Rab10, Rab12, Rab29, Rab35, and Rab43) as direct substrates for LRRK2 [13,14]. Rab proteins orchestrate membrane and vesicle trafficking [15,16]. The LRRK2 phosphorylation site (Thr72 for Rab8A, Thr73 for Rab10, and Ser106 for Rab12) for Rab proteins lies at the centre of the effector-binding switch-II motif, and is likely to exert its biological effect by altering ability of Rab proteins to interact with cognate effectors [13,14,17]. Recent work identified the RILPL1 and RILPL2 proteins, implicated in regulating ciliary membrane content [18], to specifically bind Rab8 and Rab10 via an RH2 domain, once phosphorylated by LRRK2 [14]. Phosphorylation of Rab8 and Rab10 by LRRK2 also prevents interactions with the effectors such as Rabin-8 (a GDP/GTP exchange factor) and GDI (GDP dissociation inhibitor) [13,17]. Our working model is that elevated LRRK2 activity contributes to the development of PD by inducing hyperphosphorylation of Rab proteins. This is supported by the finding that all pathogenic LRRK2 mutations significantly stimulate Rab protein phosphorylation [13,14,19,20]. Other studies have also linked disruption of Rab biology to PD and other forms of neurodegeneration (reviewed in [21]). For example, variations around Rab29 as well as disease-causing mutations in Rab39B and at least three other membrane trafficking machinery components (VPS35, VPS13C, and DNAJC6) are genetically associated with PD [2,3,22]. PINK1 also indirectly controls the phosphorylation of certain Rab GTPases including Rab8A, at a site distinct to LRRK2 (Ser111 on Rab8A) [23]. Furthermore, various Rab proteins attenuate cytotoxicity linked to aggregated α-synuclein, a factor that is associated with PD and other neurodegenerative diseases [24].

**Figure 1.**
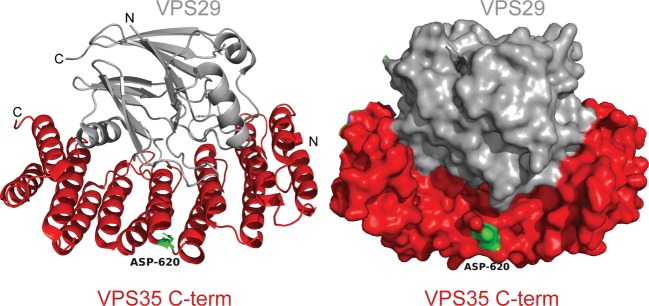
Location of the VPS35[D620N] mutation. Ribbon (left panel) and Surface (right panel) of the previously described [31] complex of VPS29 (grey) and C-terminal domain of VPS35 subunit (red). The location of the Asp620 residue that is mutated in Parkinson's is shown in green.

There is also mounting evidence that disruption of selective transport between membrane-bound organelles and the plasma membrane is a feature of PD [22,25]. A key component regulating selective transport is the retromer complex, which is made up of three proteins (VPS35, VPS29, and VPS26). This complex operates to package specific endosomal cargoes into vesicles and tubules, and deliver these to either the trans-Golgi network or to the plasma membrane [26]. In 2011, two groups reported that heterozygous changes in the gene encoding for the VPS35 cargo-binding component of the retromer complex are associated with late-onset PD [27,28]. Subsequent reports have revealed that VPS35-associated PD is overall rare with an estimated frequency of 0.4% of all PD cases and that – with over 15 000 individuals worldwide studied — the VPS35[D620N] is the only clearly pathogenic mutation characterised to date (reviewed in [29,30]). Interestingly, the structure of a VPS35 sub-complex [31] shows that the VPS35[D620N] mutation is located at the surface of the convex face of an α-helical solenoid domain (Figure 1), suggesting that the D620N mutation may impact interaction with an as yet unknown effector.

Previous work has linked LRRK2 and VPS35. This includes studies undertaken in mouse neurons as well as Drosophila that concluded that VPS35 and LRRK2 might operate in a common pathway regulating protein endolysosomal and Golgi apparatus sorting [32]. Another study found that overexpression of VPS35 or VPS26 ameliorated the pathogenic effects on the eye, as well as locomotor deficits and reduced lifespan observed in LRRK2 mutant flies [33]. Moreover, Drosophila VPS35 and LRRK2 were shown to affect the same set of synaptic vesicle processes including dopaminergic synaptic release [34]. To further investigate the interplay between VPS35 and LRRK2, we explored whether genetic manipulations of VPS35 — knock-in of the VPS35[D620N] mutation or knock-down and knock-out of VPS35 — impacted LRRK2-mediated Rab protein phosphorylation. In mouse embryonic fibroblasts (MEFs), mouse tissues (the lung, kidney, spleen, and brain) as well as patient-derived peripheral blood neutrophils and monocytes, the VPS35[D620N] mutation resulted in a marked elevation of LRRK2-mediated phosphorylation of Rab10 at Thr73. Using novel monoclonal antibodies, we demonstrate that VPS35[D620N] mutation also promotes LRRK2-mediated phosphorylation of many other Rab proteins (Rab8A, Rab10, and Rab12). Moreover, knock-out or knock-down of VPS35 also suppressed LRRK2-mediated Rab10 phosphorylation in cells. Although further work is required to pinpoint the mechanism by which VPS35 regulates LRRK2, our studies establish that VPS35 plays a major role in controlling LRRK2 protein kinase activity. Furthermore, our findings support the model that VPS35[D620N] mutation results in a gain of function and is an upstream regulator of the LRRK2 kinase pathway.

## Materials and methods

### Reagents

MLi-2 [35,36] and GSK2578215A [37] LRRK2 inhibitors were synthesised by Natalia Shpiro (University of Dundee). The PF-06447475 LRRK2 inhibitor [38] was purchased from Tocris (#5716), diisopropylfluorophosphate (DIFP) was from Sigma (#D0879), and phenylmethane sulfonyl fluoride (PMSF) was from Sigma (#78830). All peptide antigens were synthesised by ‘peptides and elephants’ (http://www.peptides.de/) and HPLC purified and stored in aliquots in sealed glass ampules in argon to avoid oxidation.

### Generation of rabbit monoclonal Rab8A (MJF-22) and phospho-Ser106 Rab12 (MJF-25) antibodies

Rabbit immunisation and rabbit antibody generation was performed by Abcam Inc. (Burlingame, CA). For the phospho-Ser106 Rab12 antibody, eight New Zealand White rabbits were immunised with target immunogens described in Table 1 using a standard protocol of five injections and two bleedings for each rabbit. Three subcutaneous injections were performed using the KLH-immunogens, followed by two subcutaneous injections using the ovalbumin-immunogens. At the time of each injection, an immunogen aliquot was thawed and combined with Complete Freund's Adjuvant (initial immunisation), or with incomplete Freund's Adjuvant (for the subsequent injections). For the total Rab8A antibody (MJF-22), six New Zealand White rabbits were immunised with a protocol of six injections and three bleedings for each rabbit. The first four injections were undertaken with the full-length human Rab8A protein and the final two injections were undertaken with a C-terminal human Rab8A peptide shown in Table 1 conjugated to KLH. Rabbits producing the best antibody were chosen for monoclonal antibody generation and were intravenously boosted with immunogen 4 days before splenectomy (for the MJFF-Rab8A programme this was undertaken using C-terminal peptide shown in Table 1). Hybridoma fusion was performed according to established protocol with minor modifications [39]. Briefly, splenocytes were harvested from the immunised rabbit and fused with rabbit plasmacytoma cells 240E-W2 [40] using PEG4000 (Sigma Chemical, St. Louis, MO) and selected by HAT (hypoxanthine, aminopterin, and thymidine). At the end of the selection, hybridoma clones growing in the original 96-well plates were transferred to 24-well plates with a medium change. Hybridoma supernatants were collected and screened for antigen binding by ELISA and western blot. Hybridomas that were specific in both assays were subcloned, expanded, and recombinant antibody prepared. For the Rab8A programme antibody, MJFF-22-74-3 was selected. For the phospho-Ser106 Rab12 programme, antibody MJFF-25-9-1 was selected. All data shown in the present study were undertaken with recombinant antibody diluted in 5% BSA (bovine serum albumin) in TBS-T. The MJFF-Rab8A and MJFF-pRab12 rabbit monoclonal antibodies will be available commercially from Abcam (www.abcam.com) in 2018. To validate the Rab12-pS106 (MJF-25-9-1) antibody, cross-reactivity was assessed using a panel of overexpressed Rab proteins as described previously [20], as well as using lysates of wild-type and Rab12 knock-out A549 cell lines described below.

**Table 1.** Antigens employed for rabbit monoclonal Rab antibodies generation.

Antibody	ABCAM project number	Antigen	Name of monoclonal antibody
Rab8A total	MJF-22	Full-length protein: MAKTYDYLFKLLLIGDSGVGKTCVLFRFSEDA FNSTFISTIGIDFKIRTIELDGKRIKLQIWDTAGQERFRTITT AYYRGAMGIMLVYDITNEKSFDNIRNWIRNIEEHASADVEKMILGNKCD VNDKRQVSKERGEKLALDYGIKFMETSAKANIN VENAFFTLARDIKAKMDKKLEGNSPQGSNQGVKITPDQQKRSSFFRCVLL Peptide: C-KMDKKLEGNSPQGSNQGVKITPDQQKRSSFFR	MJF-22-74-3
Rab12 pSer106	MJF-25	C-Ahx-AGQERFNS*ITSAYYR-amide Ac-AGQERFNS*ITSAYYR-Ahx-C	MJF-25-9-1

### Other antibodies

Mouse anti-LRRK2 C-terminus antibody was from Antibodies Incorporated (#75-253) and used at 1 µg/ml final concentration. Rabbit monoclonal antibodies for total LRRK2 (UDD3) and pS935-LRRK2 (UDD2) were purified at the University of Dundee as described previously [41] and were used at 1 µg/ml final concentration. The recombinant MJFF-pRab10 (Thr73) MJF-21-108-10 rabbit monoclonal antibody was recently described [20] and is now available from Abcam (#ab230261). The recombinant MJFF-pRab8 (Thr72) MJF-20-55-4 rabbit monoclonal antibody that detects multiple LRRK2-phosphorylated Rab proteins was also recently described [20]. All recombinant antibodies were used at 1 µg/ml final concentration. The MJFF-total Rab10 mouse antibody generated by nanoTools (www.nanotools.de) [20] was used at 2 µg/ml final concentration. Rabbit monoclonal antibodies for LRRK2 Phospho-S1292 (#ab203181), VPS35 (#ab157220), and VPS26 (#ab181352) were from Abcam and were all used at 1 : 1000 dilution. Rabbit polyclonal Rab12 antibody was from Proteintech (18843-1-AP) and was used at 1 : 500 dilution. Sheep polyclonal Rab12 was described earlier (Lis et al. [20]) and was used at 1 µg/ml. Anti-α-tubulin (#5174) antibody was from Cell Signaling Technologies and used at 1:2000 dilution. Anti-glyceraldehyde-3-phosphate dehydrogenase (GAPDH) antibody was from Santa Cruz Biotechnology (#sc-32233) and used at 50 ng/ml final concentration. Horseradish peroxidase-conjugated anti-mouse (#31450) and -rabbit (#31460) secondary antibodies were from Thermo Fisher Scientific and used at 1:2500 dilution. Goat anti-mouse IRDye 800CW (#926-32210) and IRDye 680LT (#926-68020) and goat anti-rabbit IRDye 800CW (#926-32211) secondary antibodies were from LI-COR and used at 1 : 10 000 dilution. LI-COR Quick Western Kit (IRDye® 680RD, # 926-68100) was used at 1:1000 dilution.

### Mice

Mice were maintained under specific pathogen-free conditions at the University of Dundee (U.K.). All animal studies were ethically reviewed and carried out in accordance with Animals (Scientific Procedures) Act 1986 and regulations set by the University of Dundee and the U.K. Home Office. Animal studies and breeding were approved by the University of Dundee ethical committee and performed under a U.K. Home Office project license. Mice were multiply housed at an ambient temperature (20–24°C) and humidity (45–55%) and maintained on a 12 h light/12 h dark cycle, with free access to food (SDS RM No. 3 autoclavable) and water.

### Generation of VPS35[D620N] knock-in mice

Matthew J. Farrer (The University of British Columbia) and The Michael J. Fox Foundation for Parkinson's Research generated the VPS35 D620N knock-in mice (B6(Cg)-Vps35tm1.1Mjff/J) in collaboration with Ozgene and The Jackson Laboratory. A more detailed description of how these mice was generated in currently under review and expected to be published in 2018. Briefly, a floxed ‘mini-cDNA’ was used to enable expression of the wild-type VPS35 protein prior to Cre-mediated recombination. The floxed ‘mini-cDNA’ (consisting of a splice acceptor, a sequence encoding exons 15–17, and a polyadenylation signal), with a FRT site flanked NEO cassette on the 3′ end of the mini-cDNA was inserted into intron 14–15. The construct was electroporated into C57BL/6-derived Bruce4 embryonic stem (ES) cells. Correctly targeted ES cells were injected into blastocysts and the resulting chimeric animals were tested for germline transmission. The mice were then crossed to FLP expressing mice on the C57BL/6J genetic background to remove the NEO cassette. The FLP recombinase transgene was then bred out from the line. The mice were then crossed to C57BL/6J (JAX stock No. 000664) at least once before being crossed to Cre deleter line on the B6 background (JAX stock No. 008454) to remove the floxed ‘mini-cDNA’ sequence and allow constitutive expression of the D620N mutation that had been targeted into exon 15. The mice were then bred to remove the Cre recombinase allele (https://www.jax.org/strain/023409). These mice are available from The Jackson Laboratory (JAX stock No. 023409).

Genotyping of mice was performed by PCR using genomic DNA isolated from ear biopsies. For this purpose, Primer 1 (5′-TCATTCTGTGGTTAGTTCAGTTGAG-3′), Primer 2 (5′-CCTCTAACAACCAAGAGGAACC-3′), and Primer 3 (5′-ATTGCATCGCATTGTCTGAG-3′) were used to distinguish wild-type from D620N knock-in alleles. DNA sequencing was used to confirm the knock-in mutation and performed by DNA Sequencing & Services (MRC PPU; http://www.dnaseq.co.uk) using Applied Biosystems Big-Dye version 3.1 chemistry on an Applied Biosystems model 3730 automated capillary DNA sequencers.

For experiments shown in Figure 4, 9–10-week-old age littermate-matched wild-type and VPS35[D620] knock-in mice were injected subcutaneously either with vehicle [40% (w/v) (2-hydroxypropyl)-β-cyclodextrin (Sigma–Aldrich)] or MLi-2 dissolved in vehicle at 30 mg/kg dose. Mice were killed by cervical dislocation 60 min after treatment and tissues rapidly isolated and snap-frozen in liquid nitrogen. No specific randomisation method or blinding was applied to experiments.

**Figure 2.**
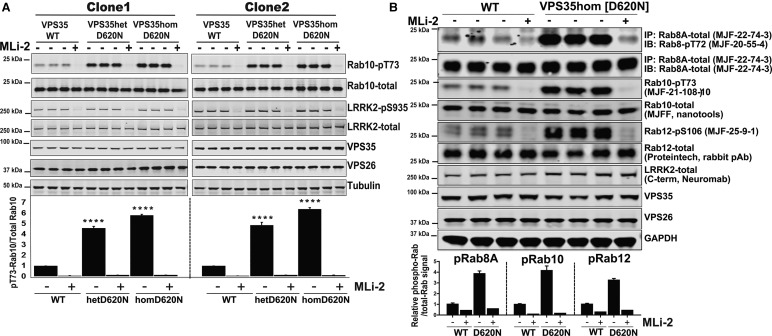
Heterozygous and homozygous VPS35[D620N] knock-in mutation in MEFs stimulates LRRK2-mediated Rab8A, Rab10, and Rab12 phosphorylation. (**A**) Wild-type VPS35, VPS35[D620N/+], and homozygous VPS35[D620N/D620N] knock-in MEFs derived from two independent sets of littermate embryos were treated ±100 nM MLi-2 inhibitor for 60 min prior to lysis. Ten to twenty micrograms of whole cell extract were subjected to quantitative immunoblot analysis with the indicated antibodies (all at 1 µg/ml primary antibody concentration), and the membranes developed using the Odyssey CLx scan Western Blot imaging system (upper panel). Similar results were obtained in three separate experiments. Immunoblots were quantified by LiCor and presented as average ± SEM (lower panel). Each lane represents cell extract obtained from a different dish of cells. There was a statistically significant difference between groups for pT73-Rab10/total Rab10 signal [*P* < 0.0001, one-way ANOVA, *F*(2, 6) = 508]. Each lane represents cell extract obtained from a different dish of cells. (**B**) As in (**A**) except that 100 µg of whole cell extract was used for immunoprecipitation of Rab8A. The immunoprecipitates were subjected to Rab8-pT72 (90% of sample) and Rab8A-total immunoblot (10% of sample). Immunoblots were quantified by LI-COR and presented as average ± SEM (lower panel).

**Figure 3.**
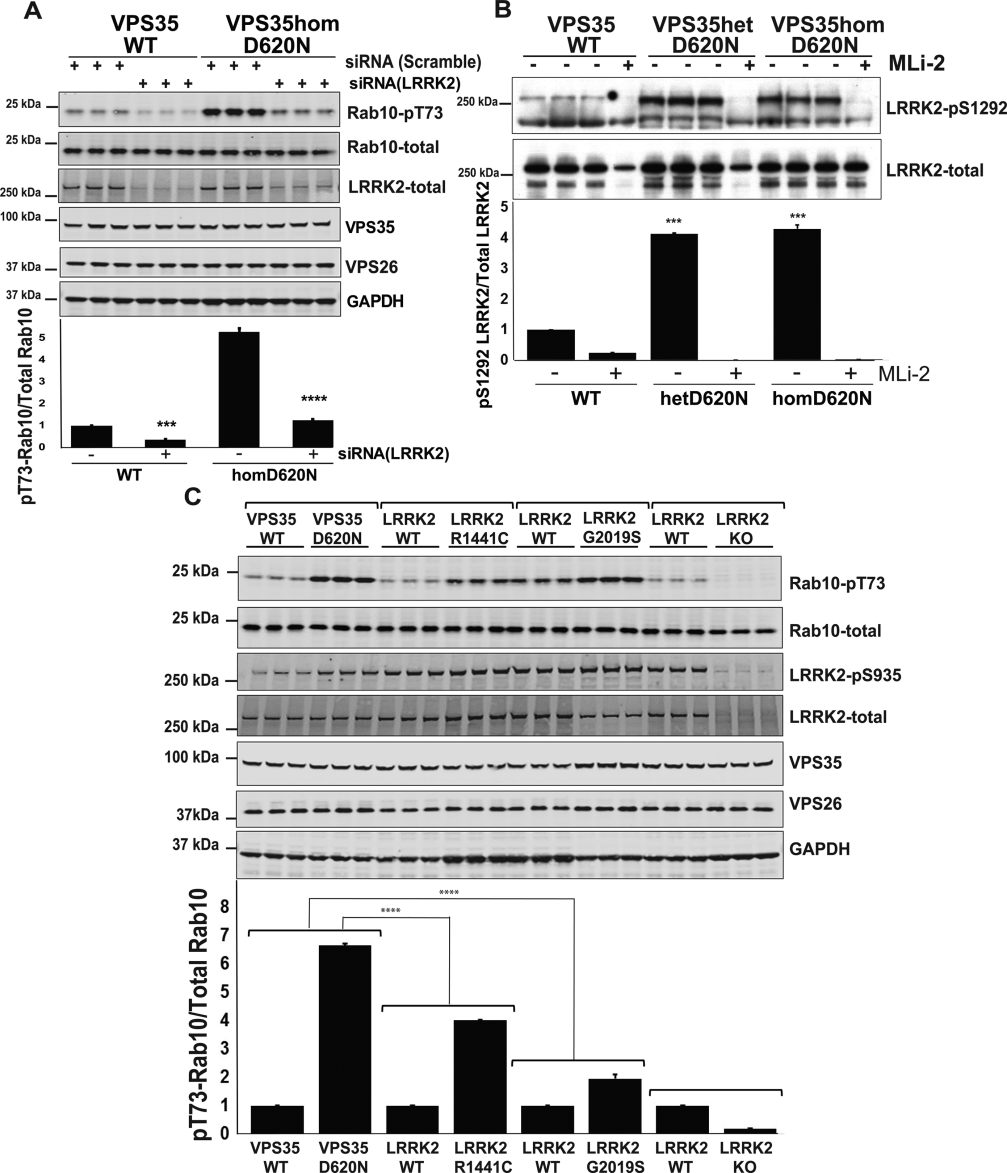
Further evidence that the VPS35[D620N] mutation enhances LRRK2 kinase activity to a greater extent than LRRK2 R1441G and G2019S pathogenic mutations. (**A**) Wild-type and VPS35 and homozygous VPS35[D620N/D620N] knock-in MEFs were transfected with the indicated pool of three Dharmacon siRNAs targeting mouse LRRK2 or scrambled siRNAs as the control. Seventy-two hour post-transfection cells were lysed. Ten to twenty micrograms of whole cell extract were subjected to quantitative immunoblot analysis with the indicated antibodies (all at 1 µg/ml primary antibody concentration), and the membranes developed using the Odyssey CLx scan Western Blot imaging system and data presented as average ± SEM. Each lane represents cell extract obtained from a different dish of cells. VPS35 WT vs. VPS35 WT siLRRK2 [*P* < 0.005, one-way ANOVA, *F*(2, 2) = 18.5], VPS35 D620N vs. VPS35 D620N siLRRK2 [*P* < 0.0001, one-way ANOVA, *F*(2, 2) = 18.5]. (**B**) Wild-type VPS35, heterozygous VPS35[D620N/+], and homozygous VPS35[D620N/D620N] knock-in were treated ±100 nM MLi-2 inhibitor for 60 min prior to lysis. The endogenous LRRK2 was immunoprecipitated from 2 mg of cell extracts and then subjected to immunoblot analysis with the indicated antibodies (upper panel). Owing to lower sensitivity of the LRRK2 Ser1292 phospho-specific antibody, immunoblots were analysed using enhanced chemiluminescent detection quantified by autoradiography (*P* < 0.0005 WT vs Het) (*P* < 0.005 WT vs. Hom). Each lane represents cell extract obtained from a different dish of cells. (**C**) The indicated matched wild-type and homozygous knock-in or knock-out MEFs derived from littermate embryos (VPS35[D620N]-the present study, R1441C [19]; G2019S-Eli-Lilly [13], LRRK2 knock-out [41]) were cultured lysed. Ten to twenty micrograms of whole cell extract was subjected to quantitative immunoblot analysis with the indicated antibodies (all at 1 µg/ml primary antibody concentration), and the membranes developed using the Odyssey CLx scan Western Blot imaging system (upper panel). Each lane represents cell extract obtained from a different dish of cells. Similar results were obtained in two separate experiments. Blots were signals quantified by LiCor and presented as average ± SEM (lower panel). There was a statistically significant difference between groups for pT73-Rab10/total Rab10 signal [*P* < 0.0001, one-way ANOVA, *F*(3, 8) = 460.5].

**Figure 4.**
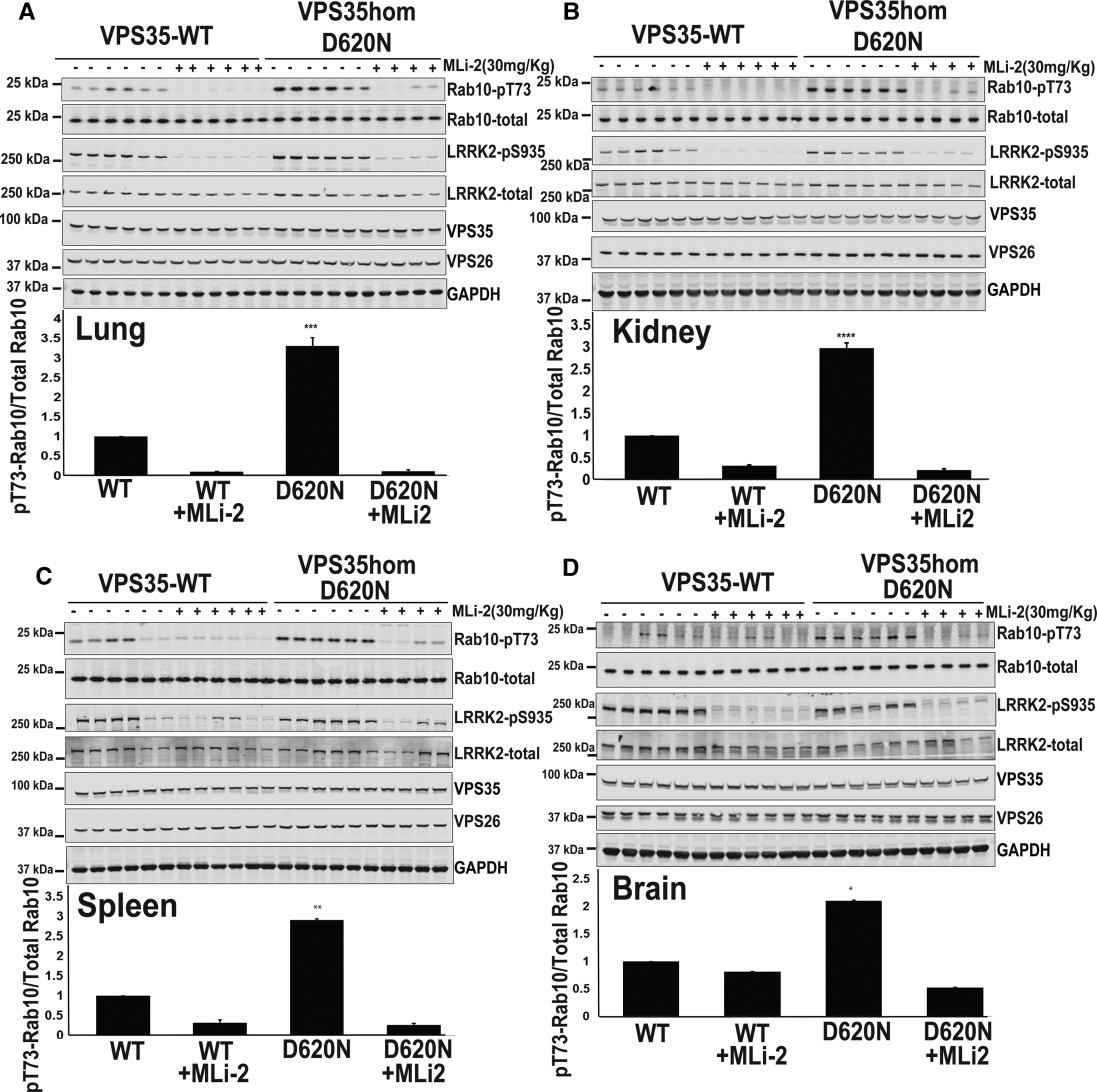
VPS35[D620N] mutation enhances LRRK2-mediated phosphorylation of Rab10 in mouse lung, kidney, and spleen. (**A**–**D**) Wild-type VPS35 and homozygous VPS35[D620N/D620N] knock-in littermate mice of 9–10 weeks of age were administered with MLi-2 (30 mg/kg) by subcutaneous injection. After 60 min, animals were killed, and the lung (**A**), kidney (**B**), spleen (**C**), and brain (**D**) extracts were generated and immunoblotted with the indicated antibodies. Ten to forty micrograms of whole cell extract were subjected to quantitative immunoblot analysis with the indicated antibodies (all at 1 µg/ml primary antibody concentration), and the membranes developed using the Odyssey CLx scan Western Blot imaging system (upper panels). Each lane represents tissues obtained from a different animal. Similar results were obtained in two separate experiments. Blots were signals quantified by LiCor and presented as average ± SEM (lower panels). There was a statistically significant difference between groups for pT73-Rab10/total Rab10 signal, Mouse Lung [*P* < 0.005, one-way ANOVA, *F*(5, 5) = 8.2], Mouse kidneys [*P* < 0.0001 one-way ANOVA, *F*(5, 5) = 4.5], Mouse Spleen [*P* < 0.005 one-way ANOVA, *F*(5, 5) = 4.99], Mouse Brain [*P* < 0.05 one-way ANOVA, *F*(5, 5) = 1.18].

### Generation of mouse embryonic fibroblasts

Wild-type, heterozygous, and homozygous VPS35[D620N] knock-in MEFs were isolated from littermate-matched mouse embryos at day E12.5 resulting from crosses between heterozygous VPS35[D620N]/wild-type mice as described previously [42]. Littermate-matched wild-type and LRRK2[R1441C] [14] and littermate-matched wild-type and LRRK2[G2019S] [20] knock-in MEFs were described recently. Littermate-matched wild-type and homozygous LRRK2 knock-out MEFs were isolated from LRRK2 knock-out mice [43] as described previously [41]. All knock-in and knock-out cell lines were verified by PCR.

### Generation of CRISPR/Cas9 knock-out of VPS35 in A549 cells

To generate VPS35 knock-out A549 cells, a modified Cas9 nickase system was used. Guides were chosen following careful transcript analysis using both NCBI and Ensembl and identified using the Sanger Centre CRISPR finder tool (http://www.sanger.ac.uk/htgt/wge/find_crisprs). Optimal sgRNA pairs were identified with a low combined off-targeting score [VPS35 KO-sgRNA1: GCCTGGTAAGAATGGAGATGT (DU57741); sgRNA2: GCTGGACCTTCACAGCCTGTA (DU57754)]. Complementary oligos with BbsI-compatible overhangs were designed for each and the dsDNA guide inserts ligated into BbsI-digested target vectors; the antisense guides (sgRNA2) were cloned onto the spCas9 D10A-expressing pX335 vector (Addgene plasmid no. 42335) and the sense guides (sgRNA1) into the puromycin-selectable pBABED P U6 plasmid (Dundee-modified version of the original Cell Biolabs pBABE plasmid). A549 cells at ∼80% confluency were co-transfected with DU57741 and DU57754 plasmids using Lipofectamine LTX according to the manufacturer's instructions, with a final amount of 6 µl Lipofectamine LTX and 3 µg of DNA per well in a 6-well plate. Twenty-four hours after transfection, the medium was replaced with a fresh medium supplemented with 2 µg/ml of puromycin. After 24 h of puromycin selection, the medium was replaced again with a fresh medium without puromycin and the cells were left to recover for 48 h before performing single-cell sorting. Wild-type A549 cells were also single-cell sorted in parallel and three single clones were selected and used as controls alongside the VPS35 knock-out clones. Cell sorting was performed using Influx cell sorter (Becton Dickinson). Single cells were placed in individual wells of a 96-well plate containing DMEM supplemented with 10% FBS, 2 mM l-glutamine, 100 units/ml penicillin, and 100 mg/ml streptomycin and 100 mg/ml Normocin (InvivoGen). After reaching ∼80% confluency, individual clones were transferred into six-well plates. After reaching ∼80% confluency, the clones were screened for VPS35 expression by immunoblotting.

### siRNA-mediated knock-down of VPS35 and LRRK2 in MEFs

Pre-designed siRNA were ordered from Dharmacon: ON-TARGETplus Mouse Vps35 (65114) siRNA — SMARTpool (# L-063309-01-0005), ON-TARGETplus Mouse LRRK2 siRNA-SMARTpool (#L-049666-00-0005), and ON-TARGETplus Non-targeting Pool (# D-001810-10-05). MEFs at 70–80% confluence were transfected with non-targeting control siRNA or VPS35 or LRRK2 siRNA at a final concentration of 50 nM using RNAiMAX transfection reagent according to the manufacturer's instructions and harvested 72 h after transfection.

### Cell culture, treatment, and lysis

A549 cells were grown in DMEM containing 10% foetal calf serum, 2 mM l-glutamine, 100 U/ml penicillin, and 100 mg/ml streptomycin. MEFs were grown in DMEM containing 10% foetal calf serum, 2 mM l-glutamine, 100 U/ml penicillin, and 100 mg/ml streptomycin supplemented with non-essential amino acids and 1 mM sodium pyruvate. All cells were grown at 37C, 5% CO_2_ in a humidified atmosphere. All cell lines were tested regularly for mycoplasma contamination. Cells were treated with LRRK2 inhibitor MLi-2 at a final concentration of 100 nM for 60 min. Cells were lysed in an ice-cold lysis buffer containing 50 mM Tris–HCl, pH 7.4, 1% (v/v) Triton X-100, 10% (v/v) Glycerol, 1 mM sodium orthovanadate, 50 mM NaF, 10 mM 2-glycerophosphate, 5 mM sodium pyrophosphate, 0.1 µg/ml microcystin-LR (Enzo Life Sciences, Switzerland), and complete EDTA-free protease inhibitor cocktail (Roche). Lysates were clarified by centrifugation at 20 800 ***g*** at 4°C for 10 min and supernatants were quantified by Bradford assay.

### Mouse tissue lysate preparation

Frozen mouse tissues were rapidly defrosted in the ice-cold lysis buffer [50 mM Tris–HCl, pH 7.5, 1% (v/v) Triton X-100, 1 mM EGTA, 1 mM sodium orthovanadate, 50 mM NaF, 10 mM 2-glycerophosphate, 5 mM sodium pyrophosphate, 0.1 µg/ml microcystin-LR (Enzo Life Sciences), 270 mM sucrose, and complete EDTA-free protease inhibitor cocktail (Sigma–Aldrich Cat # 11836170001)] and homogenised using a POLYTRON homogeniser (KINEMATICA) on ice (5 s homogenisation, 10 s interval, and 5 s homogenisation). Lysates were clarified by centrifugation at 20 800 ***g*** for 30 min at 4°C and supernatants were quantified by Bradford assay and used for subsequent immunoblot analysis.

### LRRK2 immunoprecipitation

To assess endogenous LRRK2 S1292 phosphorylation in VPS35 wild-type, D620N heterozygous, and D620N homozygous MEFS, LRRK2 was immunoprecipitated from lysates (2 mg of protein) using 4 µg of anti-LRRK2 antibody UDD3 coupled to Protein A Sepharose beads. Immunoprecipitated LRRK2 was washed three times with lysis buffer and eluted from the beads with 2× NuPAGE LDS Sample Buffer. Eluted samples were used for detecting total LRRK2 and phospho-Ser1292 LRRK2 by immunoblotting analysis. For detecting immunoprecipitated LRRK2 (phospho and total), VeriBlot secondary antibody (Abcam, #ab131366) was used instead of normal anti-rabbit IgG secondary antibody.

### A549 knock-out cell lines

The A549 Rab8A knock-out cell line has been described previously [14]. The A549 Rab12 knock-out cell line was generated using the DU54555/DU54559 constructs (available at https://mrcppureagents.dundee.ac.uk), targeting exon 3 of Rab12. Cells at ∼80% confluency were co-transfected in a six-well plate with the pair of constructs using Lipofectamine LTX reagent, with a final amount of 9 µl Lipofectamine LTX and 2.5 mg DNA per well. Twenty-four hours after transfection, the medium was replaced and fresh medium supplemented with puromycin (2 mg/ml). After 24 h selection, the medium was replaced with a medium without puromycin and the cells were left to recover for 48 h before performing single-cell sorting using an Influx cell sorter (Becton Dickinson). Single cells were placed in individual wells of a 96-well plate containing DMEM supplemented with 10% FBS, 2 mM l-glutamine, 100 units/ml penicillin and 100 mg/ml streptomycin and 100 mg/ml normocin (InvivoGen). At ∼80% confluency individual clones were transferred into six-well plates and screened for Rab12 knock-out by western blotting.

### Rab8A immunoprecipitation to assess endogenous Rab8A phosphorylation

To assess endogenous Rab8A phosphorylation, 1 µg of the recombinant MJF-22-74-3 antibody was coupled to 5 µl of Protein A Sepharose. Hundred micrograms of cell lysate were incubated for 1 h with Protein A Sepharose-antibody conjugate at 4°C with gentle agitation. The supernatants from each immunoprecipitate were retained and subjected to Rab8A immunoblot analysis in comparison with whole cell lysate to ascertain quantitative immuno-depletion of endogenous Rab8A. Immunoprecipitates were washed three times with the same buffer employed to lyse cells with. The washed beads are incubated 10 µl of 2× NuPAGE LDS Sample Buffer, vortexed and centrifuged through a Spin-X column to remove beads. The eluted sample was subjected to immunoblot analysis with both total Rab8A antibody (10% of sample) and Rab8-pT72 antibody (90% of sample). LiCor Quick Western Kit (IRDye® 680RD) was used as a secondary antibody for this analysis. To validate the immunoprecipitation efficiency, the post-IP supernatants were subjected to Rab8A immunoblotting.

### Study participants and blood sample collection

**Figure 5.**
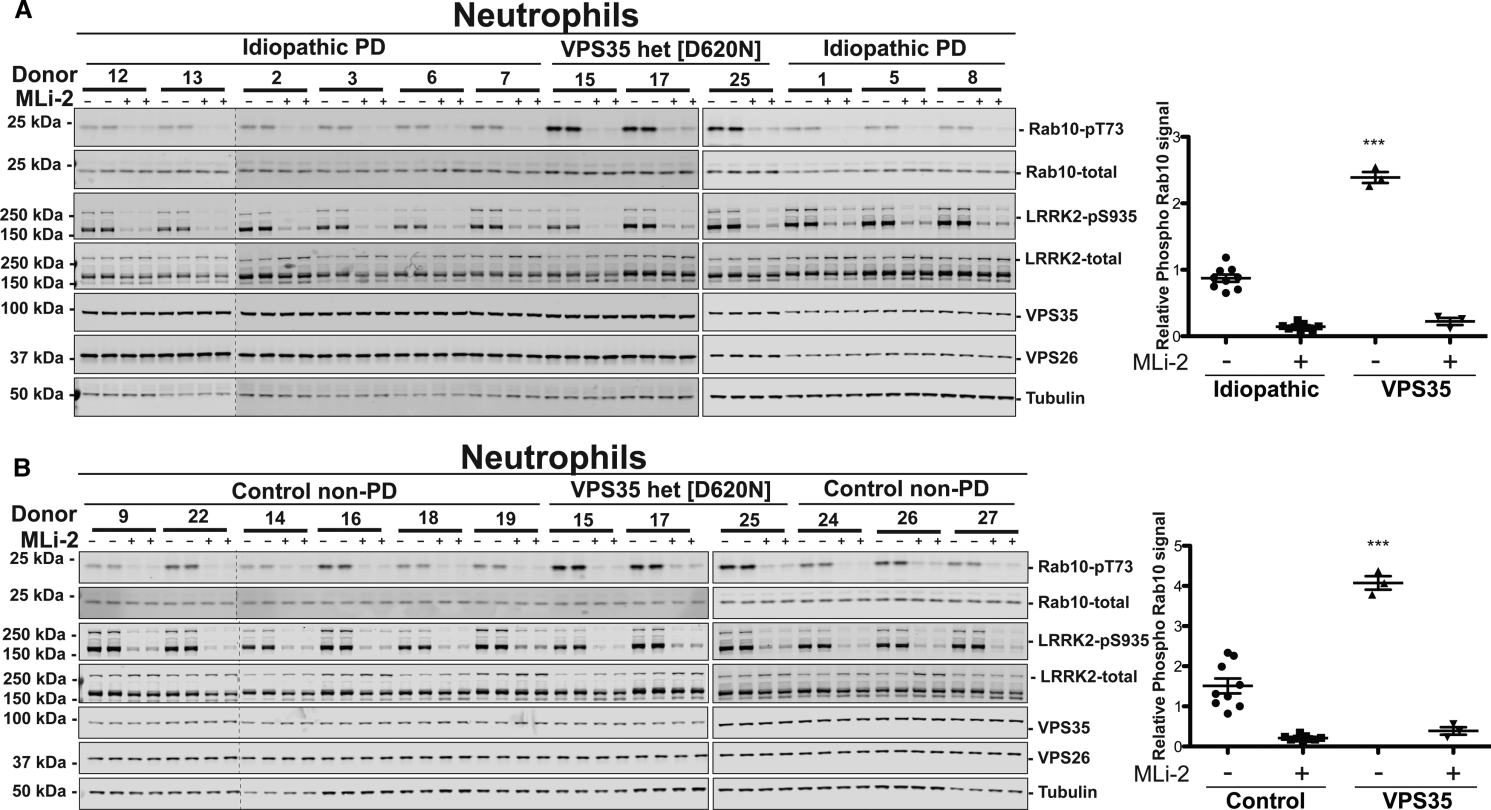
Comparing LRRK2-mediated Rab10 phosphorylation in neutrophils from control, idiopathic, and VPS35[D620N] Parkinson's patients. Neutrophils were isolated from nine age-matched idiopathic PD patients, nine non-age-matched healthy controls, and three individuals with a heterozygous VPS35[D620N] mutation with PD. Demographic and clinical data for each subject analysed are provided in Supplementary Table S1. Cells were treated with or without 200 nM MLi-2 for 30 min. Neutrophils were then lysed and 10 µg of whole cell extract subjected to quantitative immunoblot analysis with the indicated antibodies (all at 1 µg/ml primary antibody concentration), and the membranes developed using the Odyssey CLx scan Western Blot imaging system. (**A**) Samples from nine age-matched idiopathic PD patients controls and three individuals with a heterozygous VPS35[D620N] mutation with PD were analysed (left and middle panel) by immunoblotting analysis with the indicated antibodies. Samples analysed on the left hand and middle panels were generated ∼8 weeks apart and analysed on different gels. The same internal standard was run on every gel to compare the different sets of samples. Similar results were obtained in two independent immunoblot experiments of the same extracts. Immunoblots from were quantified for phospho-Thr73 Rab10/total Rab10 ratio (right panel). Data were analysed by one-way ANOVA with Tukey's multiple comparison test. Data presented as means  ±  SD; ***P < 0.0001. (**B**) As in (**A**) except neutrophil extracts from nine non-age-matched control samples were compared with the same three individuals analysed in (**A**) with a heterozygous VPS35[D620N] mutation with PD.

**Figure 6.**
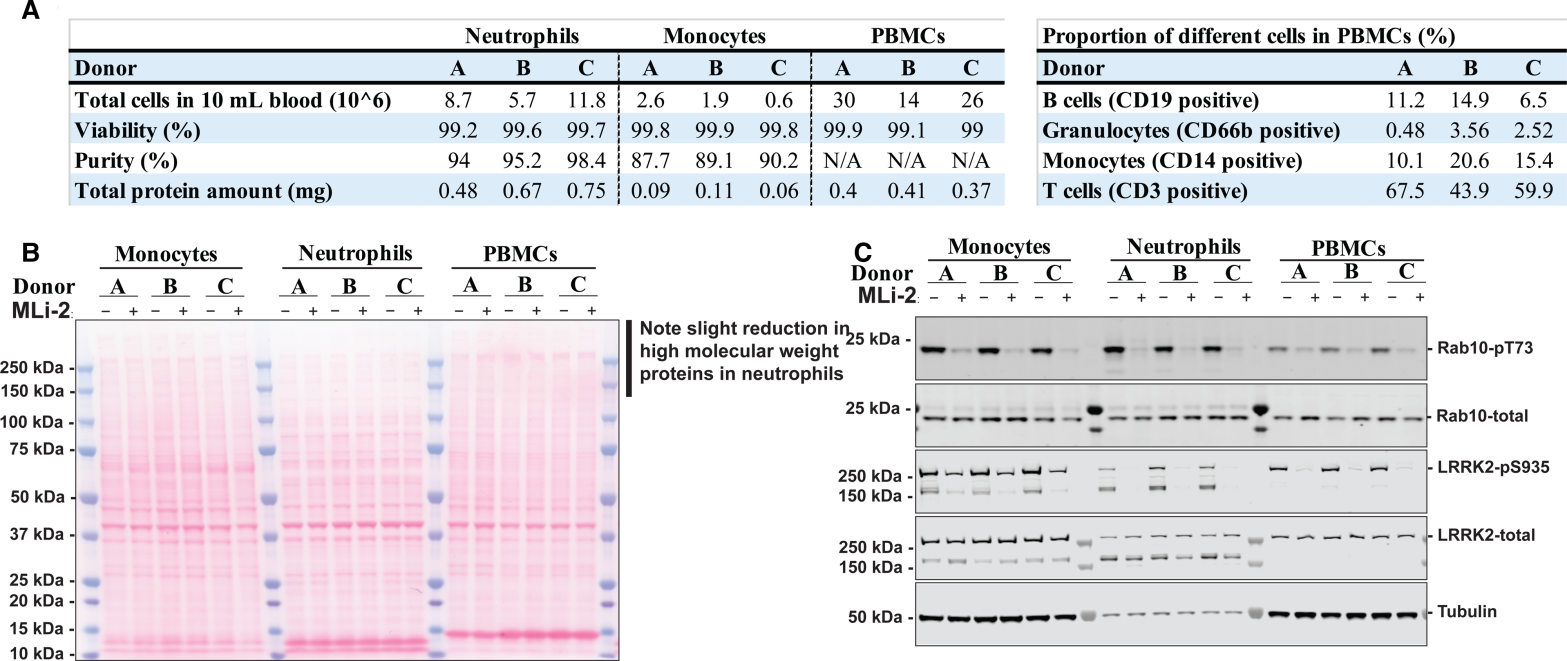
Developing an assay to interrogate LRRK2-mediated Rab10 phosphorylation in human monocytes. Monocytes, neutrophils, and PBMCs were isolated from whole blood of the same three healthy donors (termed **A**, **B**, and **C**) in parallel and treated with or without 200 nM MLi-2 for 30 min. (**A**) After gating around cells, the viability (DAPI) and purity of monocytes (CD14-positive) and neutrophils (CD66b-positive) was assessed via flow cytometer analysis. Flow cytometry was also used to assess viability and the proportion of B cells (CD19-positive), monocytes (CD14-positive), T cells (CD3-positive) and contaminating neutrophils (CD66b-positive) in each of the three PBMC preparations (Supplementary Figure S4). (**B** and **C**) Monocytes, neutrophils, and PBMCs were then lysed and 10 µg of whole cell extract subjected to SDS–PAGE and western blotting and the resulting membranes were stained with Ponceau S solution (**B**) or subjected to quantitative immunoblot analysis with the indicated antibodies (all at 1 µg/ml primary antibody concentration) and the membranes developed using the Odyssey CLx scan Western Blot imaging system (**C**).

**Figure 7.**
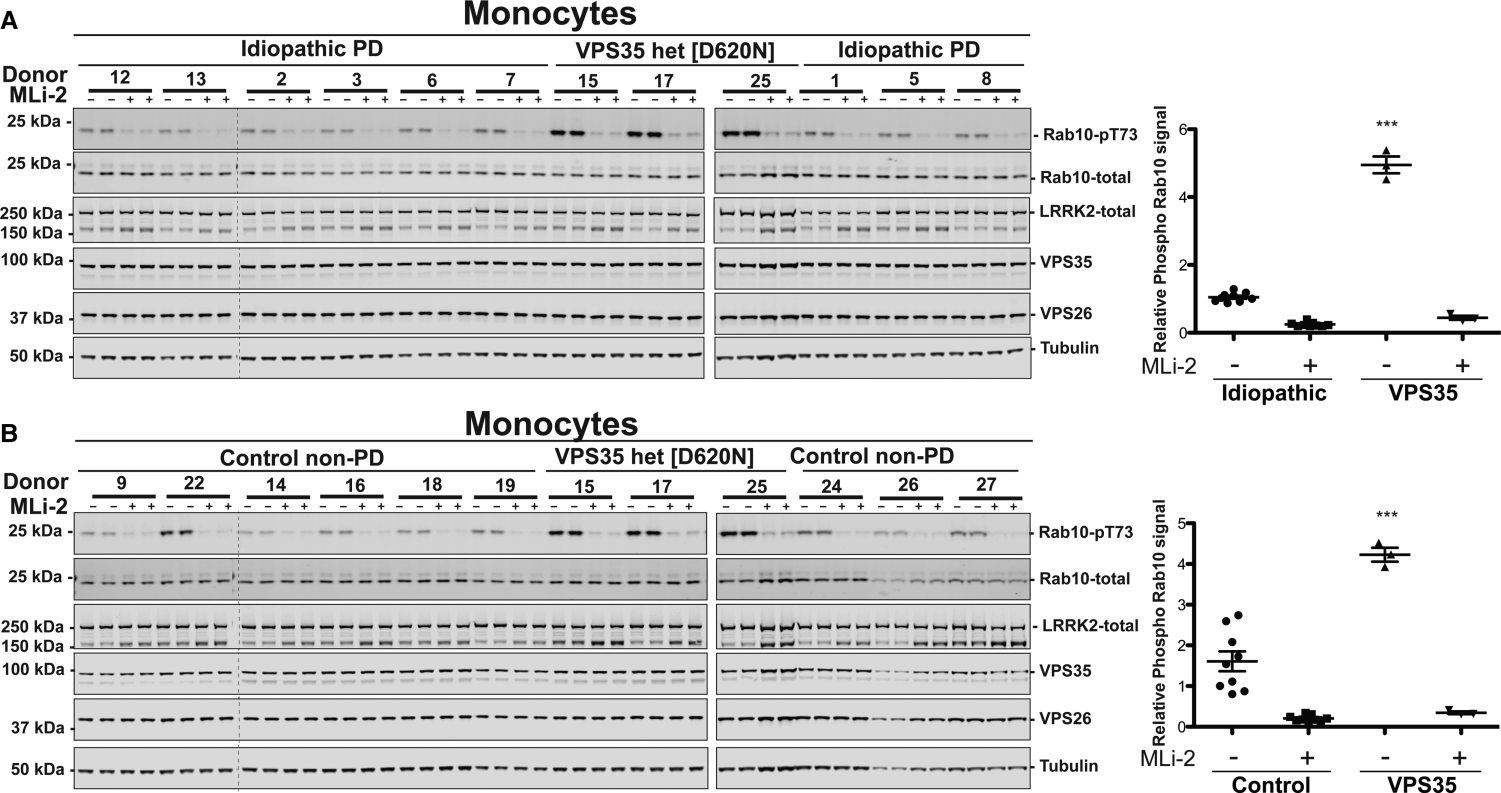
Comparing LRRK2-mediated Rab10 phosphorylation in monocytes from control, idiopathic, and VPS35[D620N] Parkinson's patients. Monocytes were isolated from nine age-matched idiopathic PD patients, nine non-age-matched healthy controls, and three individuals with a heterozygous VPS35[D620N] mutation with PD. Demographic and clinical data for each subject analysed are provided in Supplementary Table S1. Cells were treated with or without 200 nM MLi-2 for 30 min. Monocytes were then lysed and 10 µg of whole cell extract subjected to quantitative immunoblot analysis with the indicated antibodies (all at 1 µg/ml primary antibody concentration), and the membranes developed using the Odyssey CLx scan Western Blot imaging system. (**A**) Samples from nine age-matched idiopathic PD patients controls, and three individuals with a heterozygous VPS35[D620N] mutation with PD were analysed (left and middle panel) by immunoblotting analysis with the indicated antibodies. Samples analysed on the left hand and middle panels were generated ∼8 weeks apart and analysed on different gels with immunoblotting undertaken in parallel. The same internal standard was run on every gel to compare samples run on different gels. Similar results were obtained in two independent immunoblot experiments of the same extracts. Immunoblots from were quantified for phospho-Thr73 Rab10/total Rab10 ratio (right panel). Data were analysed by one-way ANOVA with Tukey's multiple comparison test. Data presented as means  ±  SD; ***P < 0.0001. (**B**) As in (**A**) except monocyte extracts from nine non-age-matched control samples were compared with the same three individuals analysed in (**A**) with a heterozygous VPS35[D620N] mutation with PD.

For setting up and validating the LRRK2 activity assay in human monocytes, we recruited volunteers from within the School of Life Sciences at the University of Dundee who kindly donated blood for the present study. Specifically, the data shown in Figure 6 are derived from three healthy volunteers from Dundee. For the clinical part of our study (in Figures 5 and 7), blood was collected with ethical approval and consent to participate was granted by the respective local ethics committees of the University of Dundee in the U.K. as well as the Medical University of Vienna in Austria. All participants provided informed consent.

### Monocyte isolation, characterisation, treatments, and lysis

Monocytes were isolated from peripheral human blood by immunomagnetic negative selection using the EasySep Human Monocyte Isolation Kit (STEMCELL Technologies, Cat# 19359) and Easy 50 EasySep Magnets (STEMCELL Technologies, Cat# 18002) following the manufacturer's protocol. Twenty millilitres of whole blood were collected in K2-EDTA BD vacutainers (Cat# BD 367525) and transferred into a 50 ml falcon tube. Additional EDTA in phosphate-buffered saline (PBS) to a concentration of EDTA at 1 mM was added (although this is not a specific requirement of the manufacturer), and tubes were gently mixed by inversion. One millilitre of ‘Isolation Cocktail’ from the monocyte isolation kit was added to the whole blood (i.e. 50 µl/ml of blood). One millilitre of resuspended RapidSpheres magnetic beads from the isolation kit was added to the blood sample (i.e. 50 µl/ml of blood), which was then gently mixed by inversion and incubated at room temperature for 5 min. The sample was then diluted to a final volume of 50 ml with 1 mM EDTA in PBS and then gently mixed by inversion of the tube. The falcon tube was next placed into the Easy 50 EasySep Magnet without lid (to avoid subsequent agitation of the sample) and incubated for 10 min at room temperature to remove non-monocyte blood cells. The supernatant containing the enriched monocyte suspension was carefully pipetted into a new 50 ml falcon tube, avoiding to disrupt the magnetic beads attached to the sides of the falcon tube that is in contact with the magnet as well as the red blood cells that accumulated at the bottom of the tube. To further purify monocytes, another 1 ml of resuspended RapidSpheres magnetic beads was added to the enriched neutrophils, and the sample was gently mixed by tube inversion and incubated at room temperature for 5 min. The tube was then placed into the EasySep Magnet without lid and after 5 min incubation at room temperature, the supernatant containing highly purified monocytes was carefully pipetted into a new 50 ml falcon tube, taking care to collect only the clear fraction. To ensure complete removal of magnetic beads from the cell mixture, the resulting monocyte suspension was placed for a final time in the magnet (without lid) and after 10 min incubation, the pure monocyte cell suspension was carefully pipetted into a new tube, ensuring that only the clear fraction was collected. The resulting isolated monocytes were centrifuged at 335 ***g*** for 5 min (acceleration and deceleration are both set to 5 using a Beckman Coulter Allegra X-15R Centrifuge) and resuspended in 10 ml RPMI 1640 media at room temperature by gentle pipetting. The cell suspension was divided equally into two tubes (each containing 5 ml), and either LRRK2 inhibitor MLi-2 (200 nM final concentration) or an equivalent volume of DMSO (0.1% v/v) was added to the media. After 30 min incubation at room temperature, monocytes were pelleted through centrifugation at 335 ***g*** for 5 min and cell pellets were lysed in the ice-cold lysis buffer containing 50 mM Tris–HCl, pH 7.5, 1% (v/v) Triton X-100, 1 mM EGTA, 1 mM sodium orthovanadate, 50 mM NaF, 10 mM 2-glycerophosphate, 5 mM sodium pyrophosphate, 270 mM sucrose, 1 µg/ml microcystin-LR, 0.5 mM DIFP (Sigma, Cat# D0879, freshly added to lysis buffer just before lysing cells), and Complete EDTA-free protease inhibitor cocktail. Samples were left on ice for 15 min to allow for efficient lysis before being clarified by centrifugation at 20 800 ***g*** at 4°C for 10 min. Supernatants were quantified by Bradford assay and used for immunoblotting analysis.

The viability and purity of monocytes after isolation was checked by flow cytometry analysis. Isolated cells were incubated with 2.5 µg human FC block (BD Biosciences, Cat# 564220) in 100 µl FACS buffer for 10 min at room temperature. FACS buffer comprised Dulbecco's PBS (Thermo Fisher Scientific, Cat# 14190094) + 1% foetal bovine serum (Thermo Fisher Scientific, Cat# 10270106). After blocking, cells were stained with the monocyte-specific cell surface marker CD14 using anti-human CD14 V500 (BD Biosciences, Cat# 561392, clone M5E2) at 1 : 20 dilution according to the manufacturer's instructions. Cells were also stained with 4′,6-diamidino-2-phenylindole (DAPI, Thermo Fisher Scientific, Cat# D1306) at 2.5 µg/ml before analysis on a FACS LSR Fortessa flow cytometer with DIVA software (BD Biosciences). Data were analysed using FlowJo software (FlowJo, LLC).

### Neutrophil and PBMC isolation, characterisation, treatments, and lysis

Neutrophils and PBMCs (peripheral blood mononuclear cells) were isolated from peripheral human blood and purity and viability and cell lysis performed as described previously [44].

### Immunoblotting analysis

Cell/tissue lysates were mixed with the NuPAGE LDS Sample Buffer (Life Technologies) supplemented with 1% (v/v) 2-mercaptoethanol and heated at 70°C for 10 min. Ten to twenty microgram samples were loaded on to NuPAGE 4–12% Bis-Tris Midi Gels (Thermo Fisher Scientific, Cat# WG1403) and electrophoresed at 130 V in the NuPAGE MOPS SDS running buffer (Thermo Fisher Scientific, Cat# NP0001-02). Proteins were then transferred onto nitrocellulose membranes (GE Healthcare, Amersham Protran Supported 0.45 µm NC) at 100 V for 90 min on ice in the transfer buffer [48 mM Tris–HCl and 39 mM glycine]. Transferred membranes were blocked with 5% (w/v) non-fat dry milk powder dissolved in TBS-T [20 mM Tris–HCl, pH 7.5, 150 mM NaCl, and 0.1% (v/v) Tween 20] at room temperature for 30 min. Membranes were then incubated with primary antibodies diluted in 5% BSA in TBS-T overnight at 4°C. After washing membranes in TBS-T, membranes were incubated at room temperature for 1 h with either HRP-labelled secondary antibody (Thermo Fisher Scientific #31480, #31460, #31430) diluted (1 : 2500) in 5% non-fat dry milk/TBS-T or with near-infrared fluorescent IRDye antibodies (LI-COR #925-68070, #925-32211) diluted (1 : 10 000) in TBS-T (without milk or BSA). After extensive washing in TBS-T, the membranes were developed either using ECL [Amersham ECL Western Blotting Detection Reagents (GE Healthcare)] or using the LI-COR Odyssey CLx Western Blot imaging system and signal quantified using the Image Studio software.

## Results

### VPS35[D620N] mutation enhances LRRK2-mediated phosphorylation of Rab10 in MEFs

To investigate whether the VPS35[D620N] pathogenic mutation [27,28] impacts on LRRK2 kinase activity, we obtained VPS35[D620N] knock-in mice made available through the Jackson laboratory (https://www.jax.org/strain/023409) by Michael J. Fox Foundation support. The acquired strain had the original mini-gene removed using Cre recombinase, to allow for the constitutive expression of the mutant VPS35[D620N] in all tissues. We first investigated LRRK2-mediated Rab10 phosphorylation by immunoblot analysis in wild-type, heterozygous, and homozygous VPS35[D620N] primary MEFs derived from E12.5 littermate embryos. This revealed that in two independent clones of homozygous VPS35[D620N] MEFS, the phosphorylation of Rab10 was strikingly elevated, ∼6-fold compared with wild-type cells (Figure 2A). Significant 4–5-fold elevation of Rab10 phosphorylation was also observed in heterozygous VPS35[D620N] MEFs. Treatment of MEFs with 100 nM MLi-2 LRRK2 inhibitor [35] reduced phosphorylation of Rab10 to nearly undetectable levels in wild-type as well as VPS35[D620N] knock-in MEFs, confirming that Rab10 phosphorylation was mediated by LRRK2 (Figure 2A). Levels of LRRK2 and Rab10 as well as VPS35 and VPS26 were similar in heterozygous and homozygous VPS35[D620N] and wild-type MEFs (Figure 2A), consistently with previous work showing that the VPS35[D620N] mutation does not impact on VPS35 and VPS26 expression levels [45–48]. Phosphorylation of LRRK2 at its Ser935 biomarker site [49] was also not significantly affected by the VPS35[D620N] mutation status (Figure 2A).

### VPS35[D620N] mutation enhances LRRK2-mediated phosphorylation of Rab8A and Rab12 in MEFs

To determine whether the VPS35[D620N] mutation enhances LRRK2-mediated phosphorylation of Rab proteins beyond Rab10, we exploited novel rabbit monoclonal antibodies that permit LRRK2-mediated phosphorylation of Rab8A and Rab12 to be assessed. To monitor Rab8A phosphorylation, we immunoprecipitated Rab8A from extracts of wild-type and homozygous VPS35[D620N] MEFs, employing a rabbit monoclonal antibody that we generated (MJF-22-74-3), capable of quantitatively immunoprecipitating endogenous Rab8A from cell extracts (Supplementary Figure S1A,B). This antibody detects human and mouse Rab8A and its selectivity was confirmed using Rab8A knock-out cell line (Supplementary Figure S1C). The Rab8A immunoprecipitates were immunoblotted with the previously described pRab8 antibody [20], revealing that Thr72 Rab8A phosphorylation is enhanced ∼4-fold higher in VPS35[D620N] knock-in compared with wild-type cells (Figure 2B). Treatment with MLi-2 LRRK2 inhibitor reduced Rab8A phosphorylation in wild-type and VPS35[D620N] knock-in cells to background levels (Figure 2B). It is important that pRab8 immunoblot analysis is undertaken in conjunction with immunoprecipitation of Rab8A, as the pRab8 antibody detects multiple LRRK2-phosphorylated Rab proteins [20].

To assess LRRK2 phosphorylation of Rab12, we employed a new rabbit monoclonal antibody capable of detecting Rab12 phosphorylated at Ser106 in cell extracts (MJF-25-9-1). This antibody is strikingly selective and does not cross-react with any other LRRK2-phosphorylated Rab proteins (Supplementary Figure S2A). We employed a CRISPR/CAS9 approach to generate Rab12 knock-out A549 cells that express LRRK2, albeit at significantly lower levels than MEFs [20]. The MJFF-pRAB12 monoclonal antibody detects phosphorylated Rab12 in wild-type but not in Rab12 knock-out A549 cells, confirming specificity (Supplementary Figure S2B). Moreover, MLi-2 treatment reduced phosphorylated Rab12 in wild-type A549 cells consistent with LRRK2 mediating phosphorylation of this residue (Supplementary Figure S2B). It should be noted that Rab12 is 4–5 kDa larger than other LRRK2-phosphorylated Rab proteins, and therefore endogenous LRRK2-phosphorylated Rab12 migrates with a notably higher molecular weight than Rab8 and Rab10. Direct immunoblotting of MEF extracts with the pRab12 antibodies indicated that Rab12 phosphorylation was elevated, ∼4-fold in homozygous VPS35[D620N] knock-in cells compared with wild-type cells (Figure 2B). Phosphorylation of Rab12 was reduced to basal levels in both wild-type and homozygous VPS35[D620N] knock-in cells following MLi-2 inhibitor treatment (Figure 2B). Further immunoblot analysis of the same cell extracts confirmed that LRRK2-mediated phosphorylation of Rab10 at Thr73 was also markedly increased in homozygous VPS35[D620N] knock-in cells (Figure 2B)

### Further evidence that elevated Rab10 phosphorylation in VPS35[D620N] knock-in MEFs is mediated by LRRK2

To verify that elevated Rab10 phosphorylation in VPS35[D620N] knock-in cells was mediated by LRRK2, we employed siRNA to knock-down LRRK2 expression by ∼80% in wild-type and homozygous VPS35[D620N] knock-in MEFs (Figure 3A). This reduced LRRK2-mediated phosphorylation of Rab10 ∼4-fold in wild-type as well as homozygous VPS35[D620N] knock-in cells, compared with cells treated with control scrambled siRNA (Figure 3A).

We also evaluated the ability of three structurally unrelated LRRK2 inhibitors namely MLi-2 [35], GSK2578215A [37], and PF-06447475 [38] to suppress elevated phosphorylation of Rab10 at Thr73 in VPS35[D620N] homozygous knock-in cells (Supplementary Figure S3). All three inhibitors induced a dose-dependent reduction in Rab10 phosphorylation that closely paralleled dephosphorylation of Ser935 on LRRK2, a widely used biomarker site for LRRK2 inactivation [49]. We estimated IC50 values for dephosphorylation of Rab10 by MLi-2 (<10 nM) [36], GSK2578215A (∼100 nM) [37], and PF-06447475 (10–30 nM) [38] that were within the range of previously reported values for Ser935 dephosphorylation. High doses of each of these inhibitors reduced Rab10 phosphorylation to basal levels (Supplementary Figure S3).

### VPS35[D620N] mutation enhances LRRK2-mediated autophosphorylation at Ser1292 in MEFs

Previous work has established that pathogenic mutations that activate LRRK2, including R1441G and G2019S, markedly elevate autophosphorylation of LRRK2 at Ser1292 [10,50]. To explore whether the VPS35[D620N] mutation enhanced its autophosphorylation at Ser1292, we immunoprecipitated LRRK2 from wild-type and VPS35[D620N] knock-in MEFs and subjected immunoprecipitates to phospho-immunoblot analysis. These experiments revealed that in both heterozygous and homozygous VPS35[D620N] knock-in MEFs, LRRK2 Ser1292 phosphorylation was markedly elevated compared with wild-type cells (Figure 3B). Consistently with Ser1292 phosphorylation mediated by LRRK2 autophosphorylation, 100 nM MLi-2 LRRK2 inhibitor treatment, suppressed Ser1292 phosphorylation to basal levels (Figure 3B). These results are consistent with the VPS35[D620N] mutation promoting activation and hence autophosphorylation of LRRK2.

### VPS35[D620N] mutation enhances LRRK2 kinase activity to a greater extent than LRRK2 R1441C and G2019S pathogenic mutations

We next undertook a side-by-side comparison of LRRK2-controlled Rab10 phosphorylation in homozygous knock-in VPS35[D620N] and previously described LRRK2[R1441C] [14] and LRRK2[G2019S] [20] knock-in MEFs (Figure 3C). For these studies, the increased phosphorylation of Rab10 in knock-in cells was compared with that of littermate wild-type cell lines generated in parallel to each of the knock-in cell lines (Figure 3C). These experiments revealed that the VPS35[D620N] mutation promoted Rab10 phosphorylation ∼6-fold, more than observed for the LRRK2[R1441C] (∼4-fold) and the LRRK2[G2019S] (∼2-fold) mutations (Figure 3C). Treatment with 100 nM MLi-2 LRRK2 inhibitor ablated Rab10 phosphorylation in all MEF lines. We also studied LRRK2 knock-out MEFs, as a further control, which as expected exhibited no Rab10 phosphorylation. Expression of LRRK2, Rab10, and retromer components were similar in all wild-type, knock-in, and knock-out MEFs (Figure 3C).

### VPS35[D620N] mutation enhances LRRK2-mediated phosphorylation of Rab10 in mouse lung, kidney, and spleen

Consistent with a previous report of a different strain of VPS35[D620N] knock-in mice generated using CRISPR/Cas9-mediated genome engineering [45], the homozygous VPS35[D620N] knock-in animals obtained via Jackson Laboratories, were born at the expected Mendelian frequency and displayed no overt phenotype at least up to 8 months of age which is the longest period that we have thus far maintained these mice. We analysed Rab10 phosphorylation in mouse tissues known to express high levels of LRRK2 (the lung, kidney, spleen, and brain) in 9–10 weeks of age littermate wild-type and VPS35[D620N] knock-in mice. For these experiments, we studied six different animals of each genotype (Figure 4). This revealed that the homozygous VPS35[D620N] mutation robustly enhanced Rab10 phosphorylation ∼3-fold in the lung (Figure 3A), kidney (Figure 4B), and spleen (Figure 4C), and 2-fold over background in the brain (Figure 4D). Phosphorylation of Rab10 was reduced to basal levels following administration of 30 mg/kg MLi-2 inhibitor, demonstrating that this was mediated by LRRK2. Expressions of LRRK2, Rab10, and retromer components were similar in all wild-type and homozygous VPS35[D620N] knock-in tissues (Figure 4).

### Comparing LRRK2-mediated Rab10 phosphorylation in neutrophils from control, idiopathic, and VPS35[D620N patients with PD

To address the question of whether LRRK2 kinase activity is elevated in PD patients with VPS35[D620N] mutation, we assessed Rab10 phosphorylation in neutrophils derived from peripheral blood from three VPS35[D620N] PD patients using a recently described method [44]. In parallel, we also prepared neutrophils from nine age-matched patients with idiopathic PD carrying no known pathogenic mutation (Figure 5A) as well as nine non-age-matched healthy controls (Figure 5B). Demographic and clinical data for each subject are presented in Supplementary Table S1. Prior to cell lysis, neutrophils were treated in the absence or presence of 200 nM MLi-2 LRRK2 inhibitor for 30 min to ensure that observed Rab10 phosphorylation was LRRK2 dependent. Quantitative immunoblot analysis revealed a striking ∼3-fold elevation of Rab10 phosphorylation in neutrophils derived from each of the three PD patients harbouring VPS35[D620N] compared with the average of all other patients with idiopathic disease (Figure 5A) or control samples (Figure 5B). Phosphorylation of Rab10 protein was higher in each of the three VPS35[D620N] patients' neutrophils than observed in any of the other idiopathic or control samples (Figure 5). Rab10 total protein levels were remarkably constant between the different donors (Figure 5). MLi-2 treatment suppressed Rab10 phosphorylation to low background levels in all neutrophil samples confirming that Rab10 phosphorylation was mediated by LRRK2 (Figure 5). Levels of the retromer complex components VPS35 and VPS26 were also very similar in neutrophil samples isolated from control, VPS35[D620N] mutation, and idiopathic subjects (Figure 5). As observed in previous work suggesting that LRRK2 was significantly proteolysed in neutrophil extracts [44], immunoblot analysis revealed two species of LRRK2 migrating at ∼286 kDa (full-length) and a more abundant ∼170 kDa species (Figure 5).

### Developing an assay to interrogate LRRK2-mediated Rab10 phosphorylation in human monocytes

We next explored the feasibility of studying LRRK2 signalling in monocytes, as these are the only peripheral human blood cells other than neutrophils that express high levels of LRRK2 (∼1 × 10^5^ copies per cells) as well as Rab10 (2–4 × 10^6^ copies per cells) [51]. Another potential advantage of monocytes over neutrophils is that they do not express high levels of elastase protease activity that leads to significant proteolysis of LRRK2. As a protocol to isolate monocytes for the assessment LRRK2 signalling had not been previously described, we first developed a method to isolate monocytes from peripheral blood exploiting an immune-magnetic negative isolation approach, in which all non-monocytic cells are targeted for removal with antibody complexes recognising unwanted cells, including red blood cells, neutrophils, and platelets, leaving only monocytes in the supernatant (see Materials and methods). Flow cytometry analysis with the V500 Mouse Anti-Human CD14 monocyte marker revealed that the purity of monocytes isolated from each volunteer was 87–90% and viability of cells was ∼99% assessed with DAPI staining (Figure 6A and Supplementary Figure S4). From 20 ml of blood, we obtained between 0.06 and 0.11 mg of total protein from healthy donors that is ∼5- to 10-fold less than the amount of protein obtained from neutrophils isolated from the same donor (Figure 6A). This is expected as monocytes are much less abundant in human blood than neutrophils. Nevertheless, the amount of protein obtained from each monocyte preparation is sufficient for a handful of immunoblot analyses that typically require 10–20 µg protein per gel lane.

To demonstrate the feasibility of studying LRRK2 signalling in monocytes, we isolated monocytes, neutrophils, and PBMCs from the same three healthy volunteers (Figure 6 and Supplementary Figure S4). Monocytes, neutrophils, and PBMCs from each subject prior to cell lysis were treated in the presence or absence of 200 nM MLi-2 for 30 min. Quantitative immunoblot analysis revealed robust and similar levels of LRRK2-mediated phosphorylation of Rab10 were observed in monocytes as well as neutrophils, with lower levels seen in PBMCs (Figure 6C). In PBMCs, LRRK2 expression is lower than observed in monocytes and neutrophils (Figure 6C). As was predicted, immunoblot analysis revealed significantly higher levels of full-length LRRK2 in monocytes compared with neutrophils and PBMCs (Figure 6C). Ponceau S staining of immunoblots confirms a slight reduction in high molecular mass protein expression in neutrophils relative to monocytes and PBMCs likely due to enhanced proteolysis (Figure 6B). As predicted, full-length LRRK2 was much more abundant in monocyte extracts compared with neutrophils (Figure 6).

### Comparing LRRK2-mediated Rab10 phosphorylation in monocytes from control, idiopathic, and VPS35[D620N] patients with PD

We next isolated monocytes from the same three VPS35[D620N] patients as well as nine age-matched idiopathic PD patients and nine non-age-matched non-PD controls who provided neutrophils for the experiments shown in Figure 5. Monocytes from each subject prior to cell lysis were also treated in the presence or absence of 200 nM MLi-2 for 30 min. Quantitative immunoblot analysis of monocyte lysates revealed a striking ∼3-fold elevation of Rab10 phosphorylation in the three VPS35[D620N] patients compared with idiopathic patients (Figure 7A) or controls (Figure 7B). MLi-2 treatment suppressed Rab10 phosphorylation to background levels in all monocyte samples confirming that Rab10 phosphorylation was mediated by LRRK2. Levels of total Rab10 protein and the retromer complex components VPS35 and VPS26 were very similar in monocytes derived from different donors (Figure 7).

### Knock-out and siRNA-mediated knock-down of VPS35 suppress LRRK2 kinase activity

**Figure 8.**
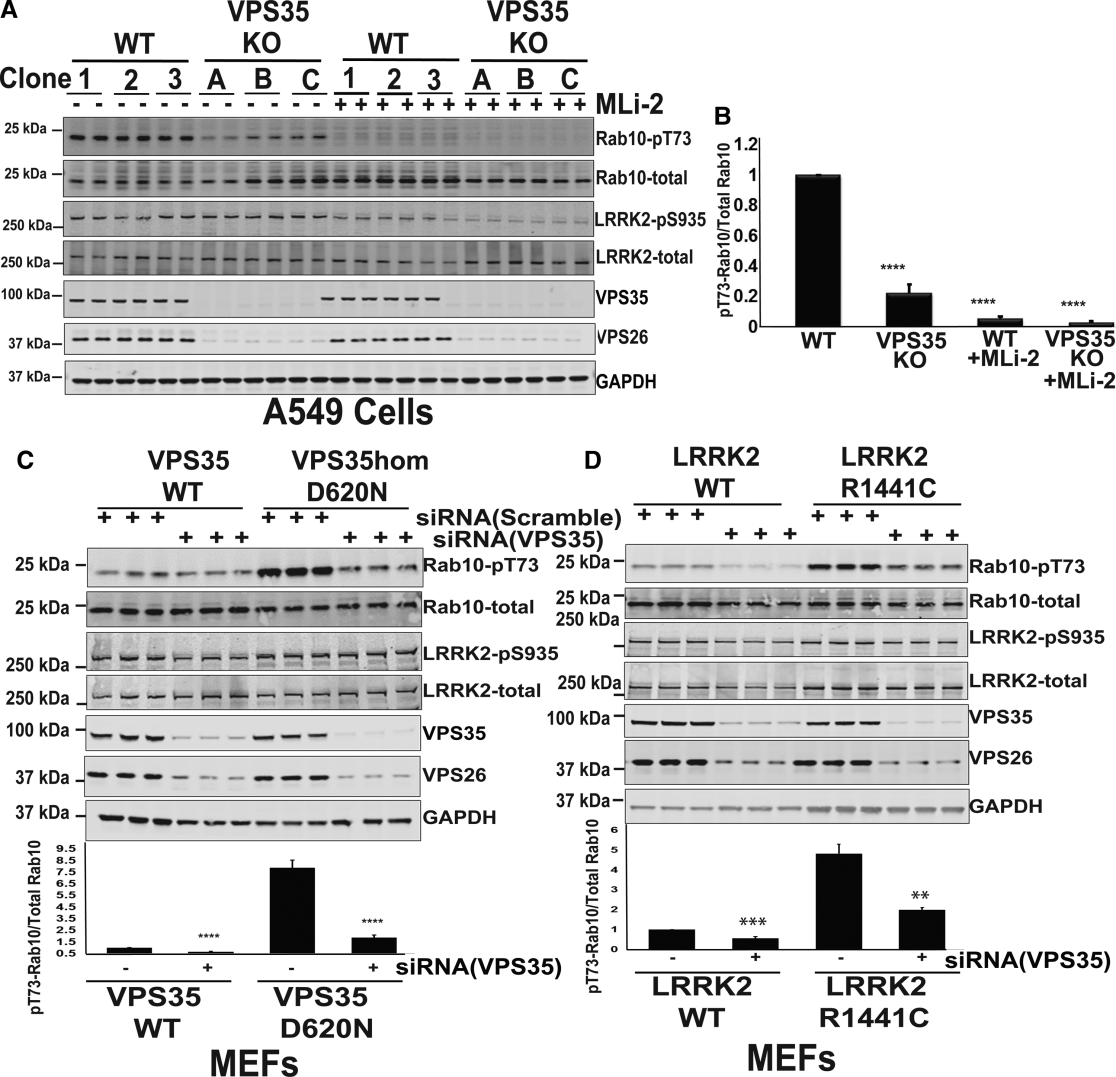
Knock-out and siRNA-mediated knock-down of VPS35 suppress LRRK2 kinase activity. (**A**) Three independent wild-type (WT1, WT2, and WT3) VPS35 and three independent CRISPR/CAS9 VPS35 knock-out (KO-1, KO-2, and KO-3) A549 cells that had all been through the identical single-cell sorting and expansion procedure were treated ±100 nM MLi-2 inhibitor for 60 min prior to lysis. Ten micrograms of whole cell extract subjected to quantitative immunoblot analysis with the indicated antibodies (all at 1 µg/ml primary antibody concentration), and the membranes developed using the Odyssey CLx scan Western Blot imaging system. Similar results were obtained in three separate experiments. (**B**) Immunoblots were quantified by LiCor and presented as average ± SEM. There was a statistically significant difference between groups for pT73-Rab10/total Rab10 signal [*P* < 0.0001, one-way ANOVA, *F*(3, 20) = 361]. (**C**) Wild-type and VPS35 and homozygous VPS35[D620N/D620N] knock-in MEFs were transfected with the indicated pool of three Dharmacon siRNAs targeting mouse VPS35 or scrambled siRNAs as the control. Seventy-two hours post-transfection cells were lysed and immunoblotted with the indicated antibodies (upper panel). Similar results were obtained in three separate experiments. Immunoblots were quantified by LiCor and presented as average ± SEM. VPS35 WT vs. VPS35 WT siVPS35 [*P* < 0.0001, one-way ANOVA, *F*(2, 2) = 7.8], VPS35 D620N vs. VPS35 D620N siVPS35 [*P* < 0.0001, one-way ANOVA, *F*(2, 2) = 7.3]. (**D**) As in (**C**) except that wild-type LRRK2 and homozygous LRRK2 [R1441C/R1441C] knock-in MEFs were used. LRRK2 WT vs. LRRK2 WT siVPS35 [*P* < 0.005, one-way ANOVA, *F*(2, 2) = 3.4], LRRK2 R1441C vs. LRRK2 R1441C siVPS35 [*P* < 0.05, one-way ANOVA, *F*(2, 2) = 281].

To analyse the role of wild-type VPS35 in regulating the activity of wild-type LRRK2, we deployed a CRISPR/CAS9 genome-editing approach to knock-out VPS35 in lung A549 cells that express significant levels of LRRK2. We analysed three independent VPS35 knock-out cell lines as well as three independent wild-type cell lines that had all been single-cell sorted and expanded in parallel. Strikingly, in all the VPS35 knock-out cell lines, levels of LRRK2-mediated Rab10 phosphorylation were reduced 4–5-fold compared with wild-type cells (Figure 8A,B). Consistently with previous work [52], knock-out of VPS35 led to a substantial reduction in VPS26, which is another core component of the retromer complex (Figure 8A).

We also employed siRNA to knock-down VPS35 expression by ∼80% in wild-type (Figure 8C,D), VPS35[D620N] (Figure 8C), and LRRK2[R1441C] (Figure 8D) knock-in MEFs. This reduced LRRK2-mediated phosphorylation of Rab10 in wild-type (∼2-fold), VPS35[D620N] (∼5-fold), and LRRK2[R1441C] (∼2.5-fold), compared with cells treated with control scrambled siRNA (Figure 8C,D). As expected, partial knock-down of VPS35 also lowered expression of VPS26 (Figure 8C,D).

## Discussion

Our data indicate that VPS35 lies upstream of LRRK2 and plays a role in regulating LRRK2 kinase catalytic activity. This is based on the following findings: the pathogenic, PD causing VPS35[D620N] knock-in mutation stimulates LRRK2-mediated phosphorylation of at least 3 Rab proteins (Rab8A, Rab10, and Rab12) in MEFs (Figure 2) as well as enhancing Rab10 phosphorylation in mouse tissues that express LRRK2 (the lung, kidney, spleen, and brain) (Figure 4). In MEFs, we also demonstrate that the VPS35[D620N] knock-in mutation markedly enhances autophosphorylation of LRRK2 at Ser1292, consistent with LRRK2 becoming activated (Figure 3B). Further evidence that the VPS35[D620N] mutation stimulates LRRK2 activity comes from data derived from human peripheral blood cells from three patients with VPS35-associated PD bearing the VPS35[D620N] mutation: Rab10 phosphorylation is markedly elevated in neutrophils (Figure 5) as well as monocytes (Figure 7) isolated from VPS35[D620N] PD patients in comparison with those from healthy donors or age-matched idiopathic PD patients. We also demonstrate that siRNA knock-down of LRRK2(Figure 3A) or treatment with MLi-2 (Figures 2–8) as well as two structurally unrelated LRRK2 inhibitors (PF-06447475 and GSK2578215A) (Supplementary Figure S3) markedly inhibit Rab10 phosphorylation in VPS35[D620N] knock-in cells. This provides strong evidence that elevated Rab10 phosphorylation induced by the VPS35[D620N] mutation is indeed mediated by LRRK2.

While further studies involving greater numbers of VPS35[D620N] patients are needed to confirm that the VPS35[D620N] mutation stimulates Rab protein phosphorylation in humans, we are intrigued by the significant increase in Rab10 phosphorylation observed in peripheral blood cells from the three PD patients carrying the VPS35[D620N] mutation. In addition to studying further patients with VPS35-associated PD, it would be important to analyse phosphorylation of Rab10 as well as other Rab proteins in pre-symptomatic carriers of the VPS35[D620N] mutation. Further evidence supporting the notion that VPS35 lies upstream of LRRK2 comes from the finding that CRISPR/CAS9 knock-out of VPS35 substantially reduces the activity of wild-type LRRK2 in A549 cells (Figure 8A,B). Moreover, siRNA-mediated knock-down of VPS35 significantly reduced elevated LRRK2-mediated phosphorylation of Rab10 in LRRK2[R1441C] and VPS35[D620N] knock-in MEFs (Figure 8C,D).

The finding that VPS35 lies in a common pathway with LRRK2 is consistent with previous work that is outlined in the introduction [32–34]. Taken together, these observations provide further evidence that a significant number of proteins encoded by genes mutated in genetic PD are likely to form part of common signalling networks. Perhaps the best characterised connection between two PD-encoded proteins discovered to date is between the PINK1 kinase and Parkin E3 ligase, where loss-of-function mutations cause autosomal recessive, young onset PD [53]. PINK1 directly phosphorylates and activates Parkin E3 ligase activity in response to agents that induce mitochondrial depolarisation, which plays a vital role in regulating mitochondrial quality control in neurons [54,55]. Another example for the cross-talk between signalling pathways in PD is the interaction between the protein encoded by RAB29 gene (located within the PARK16 locus) and LRRK2 [32,56–58]. Recent work also reveals that Rab29 activates LRRK2 through an as yet unknown mechanism, by recruiting it to the trans-Golgi network [10–12]. It will be critical to determine whether other monogenetic PD-encoded proteins comprise upstream or downstream components of the LRRK2 signalling pathway.

We found that the heterozygous VPS35[D620N] mutation was sufficient to markedly activate LRRK2-mediated Rab10 phosphorylation 4–5-fold in MEFs, only slightly less than the homozygous mutation, that activated LRRK2 ∼ 4–6-fold (Figure 2A). The heterozygous VPS35[D620N] mutation also enhanced Ser1292 autophosphorylation similarly to the homozygous mutation (Figure 3B). The finding that LRRK2-mediated Rab10 phosphorylation is significantly elevated in neutrophils (Figure 5) and monocytes (Figure 7) derived from patients with PD associated with a single copy of the VPS35[D620N] allele confirms that the heterozygous mutation is sufficient to potently activate LRRK2 kinase activity in humans. Our results support that the VPS35[D620N] mutation results in a gain of function that leads to the activation of the LRRK2 signalling pathway. Much previous work has also suggested that the VPS35[D620N] mutation acts in an autosomal dominant manner (Reviewed in [29]).

**Figure 9.**
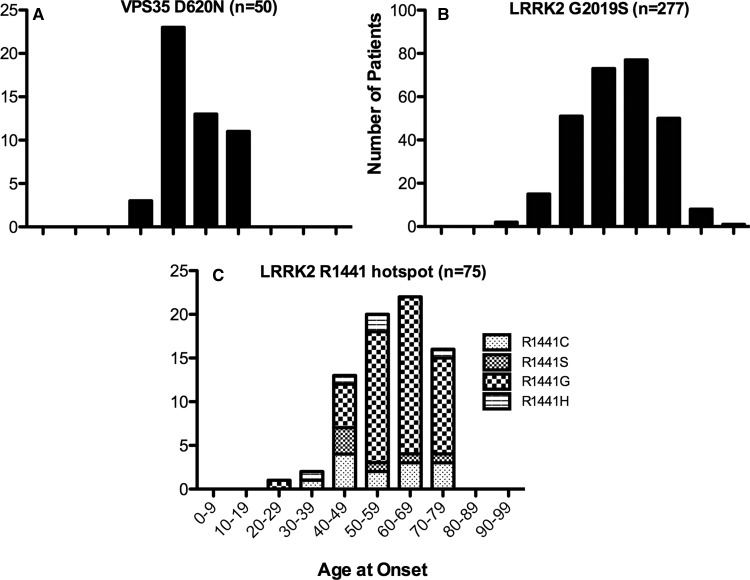
The age of onset of Parkinson's in patients with pathogenic VPS35[D620N], LRRK2[G2019S], and LRRK2[ROC–COR] mutations using data available from the MDS database. The age at onset (AAO) of PD patients with pathogenic mutations in (**A**) VPS35[D620N] (*n* = 50), (**B**) LRRK2[G2019S] (*n* = 277), and (**C**) LRRK2[ROC–COR mutations encompassing R1441C [*n* = 13], R1441S [*n* = 6], R1441G [*n* = 50], and R1441H [*n* = 5] as listed in the MDS gene database (http://www.mdsgene.org/) [58]. For R1441H, we included three additional patients published elsewhere and not included in the MDS gene database (1 patient each with AAO 32 and 57 [60] as well as 1 with AAO 59 [61]). For R1441G, we also included 49 additional cases not listed in the MDS gene database and whose AAO had previously been reported as a mean 61.7 ± 8.5 years; range 44–80 years [63]. The authors of the later study kindly made the information for the individual AAO available to us.

Another potentially significant observation is that the homozygous VPS35[D620N] mutation activated LRRK2, based on assessing Rab10 phosphorylation, to a significantly greater extent (∼6-fold) than LRRK2[G2019S] (∼2-fold) or LRRK2[R1441C] (∼4-fold) (Figure 3C). If the degree of LRRK2 activation is relevant for pathogenesis of PD, a major prediction from our studies is that VPS35[D620N] carriers may develop PD at an earlier age than carriers with pathogenic LRRK2 mutations, especially the G2019S mutation. This is currently difficult to assess rigorously due to the rarity of VPS35[D620N]-associated PD. Most studies refer to VPS35[D620N] as ‘late onset’ (reviewed [29]), while only one study comparing 35 unrelated PD cases of VPS35[D620N] concluded that the mean age at onset was 51.4 years (interquartile range, 45–59 years) [26]. Interestingly, analysis of the Movement Disorder Society (MDS) database [59], which contains 50 VPS35[D620N] patients, reveals that the largest group of patients, namely 23, were diagnosed with PD in the 5th decade, with only 13 diagnosed in the sixth decade (Figure 9A). In contrast, for LRRK2 G2019S carriers of which there are 277 in the MDS database, the largest groups of patients were diagnosed in sixth (73) and seventh decade (77) (Figure 9B). Additionally, the penetrance of the G2019S mutation is relatively low (as low as 24%) and most carriers may never develop disease [3,60]. Interestingly, one study noted that age of PD onset in LRRK2 p.G2019S carriers was younger and penetrance greater in North Africa versus Norway PD patients [60]. Consistent with a more pronounced effect on stimulating LRRK2 kinase activity, the R1441G LRRK2 mutation appears to be more penetrant (up to 95% in later life) [3,60]. We have pooled the low number of LRRK2 patients with known pathogenic hotspot mutations at Arg1441 in the ROC–COR GTPase domain listed in the MDS database (R1441C [*n* = 13], R1441S [*n* = 6], R1441G [*n* = 50], and R1441H [*n* = 5], Figure 9C) [52]. For R1441H, we also included three additional patients published elsewhere [61,62]. Additionally, the authors of a previously published paper that reported the mean age at onset and range of 49 patients with R1441G-associated PD, kindly provided us with the age at onset for each individual [63]; data that we also included in Figure 9C. The age at onset of the LRRK2 ROC–COR mutation patients does appear to be similar to that of the G2019S, which lies in the kinase domain, but possibly slightly younger. Owing to the rarity of VPS35-associated PD, there are insufficient data to reliably estimate the penetrance of the VPS35[D620N] mutation. However, our prediction is that this would be more similar to LRRK2[R1441G] carriers than LRRK2[G2019S].

**Figure 10.**
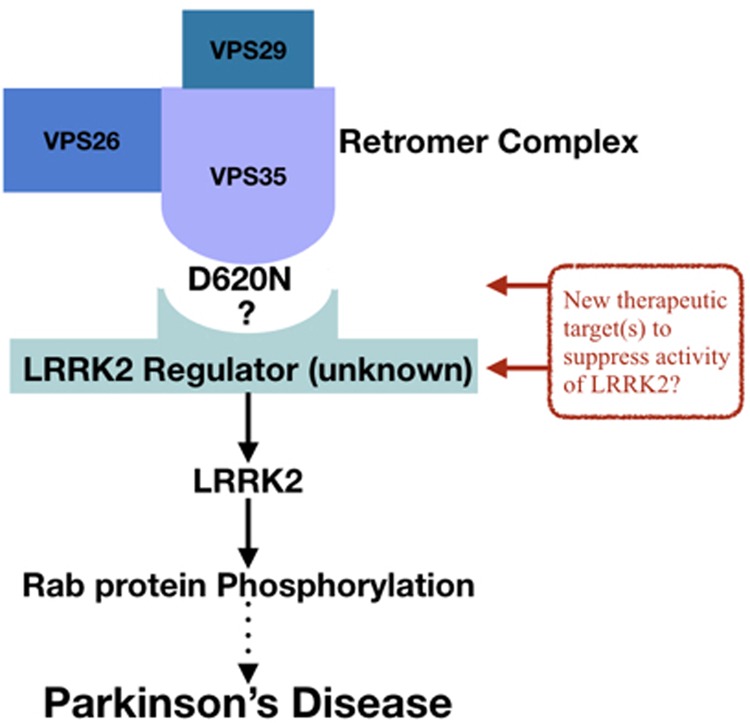
Schematic summary showing how VPS35 might regulate LRRK2 and LRRK2 protein kinase activity and hence PD. The mechanism by which LRRK2 is activated *in vivo* has not been elucidated. It is also not known what maximal activation of LRRK2 kinase activity is achievable. It is thought that the LRRK2 pathogenic mutations cause only a partial activation of LRRK2. Mutations in the kinase domain, such as G2019S, induce a moderate ∼2-fold activation of LRRK2, perhaps by stabilising the active confirmation of the kinase fold. Pathogenic mutations in the ROC/COR domain of LRRK2, such as R1441G/C, increase LRRK2 kinase activity ∼3–4-fold. Three recent papers [10–12] indicate that the ROC/COR mutations, promote recruitment of LRRK2 to the Rab29 protein that is located at the Golgi, which leads LRRK2 activation through an as yet unknown mechanism. VPS35 and Rab29 could operate in the same pathway with LRRK2 [32]. Our work shows that the VPS35[D620N] mutation through an unknown pathway is inducing a higher 5–6-fold activation of LRRK2 than achieved with the known pathogenic mutations. Indeed, this is supported by age-of-onset data shown in Figure 9 that suggest that Parkinson's patients with VPS35[D620N] develop the disease at a younger age than those with LRRK2 mutations. In future work, it would therefore be vital to define the mechanism by which VPS35[D620N] is activating LRRK2 and explain why VPS35[D620N] mutations induce a greater activation of LRRK2 than observed with the LRRK2 pathogenic mutants. We propose that by understanding this mechanism it might be possible to develop compounds that target the retromer complex that suppress activation of LRRK2.

In future work, it will be essential to decipher the mechanism by which VPS35 regulates LRRK2 and how the VPS35[D620N] mutation enhances LRRK2 kinase activity. As the D620N mutation lies on the surface of VPS35, it is possible that it suppresses or potentiates the interaction of VPS35 with a specific interactor(s) that influences LRRK2 localisation and/or catalytic activity. It is also possible that VPS35 could directly interact with LRRK2. Indeed, one study reported that in SH-SY5Y cells as well as LRRK2 transgenic mouse brain, LRRK2 and the VPS35 subunit could be co-immunoprecipitated [32], although this was not observed in another study [34]. Several hundred proteins that co-immunoprecipitate with LRRK2 have been catalogued in many elegant proteomic studies, but to our knowledge the retromer complex components were not highlighted in these [56,64]. Another report suggested that Rab29 also operates in a common pathway with VPS35 and LRRK2, which would be important to explore further [32]. The VPS35[D620N] mutant does not associate with the WASH complex as effectively as wild-type VPS35, contributing to trafficking defects [48,65]. In future work, it would be essential to explore the role that the WASH complex as well as other retromer effectors plays in regulating the LRRK2 pathway. Above all, it would be critical to find out whether it is possible to identify specific protein(s) that interact differentially with the wild-type and mutant form of LRRK2 and determine whether these proteins play a role in regulating LRRK2 activity and function (Figure 10). This work could lead to the uncovering of new regulators relevant to PD.

From a clinical point of view, there is great interest in exploring the therapeutic benefit of LRRK2 inhibitors in PD [66]. One company (Denali Therapeutics) has initiated a Phase 1 clinical trial with several others understood to be at a late stage in preclinical assessment of diverse inhibitors [67]. Our work raises the possibility of elaborating inhibitors that target VPS35 that could suppress the activation of LRRK2 (Figure 10). Identifying the molecular mechanism by which VPS35 activates LRRK2 will be critical, as this may provide new ways to suppress activation of LRRK2 kinase pathway. This could also lead to the discovery of new biomarker(s) that operate between VPS35 and LRRK2 that are relevant for assessing PD. This mechanistic analysis would ultimately provide a tractable framework to better understand underlying biology of the neurodegenerative process occurring in patients with genetic, but also idiopathic PD. For now, there is a rational that patients with VPS35[D620N]-associated PD might benefit from future LRRK2 inhibitor treatment, in particular if the disease mechanism as well as the possibly earlier age of onset and possible higher penetrance of VPS35[D620N] PD is indeed driven by hyperactivation of the LRRK2 kinase pathway.

## Abbreviations

BSA: bovine serum albumin
DAPI: 4′,6-diamidino-2-phenylindole
DIFP: diisopropylfluorophosphate
ES: embryonic stem
LRRK2: Leucine-Rich Repeat protein kinase-2
MDS: Movement Disorder Society
MEFs: mouse embryonic fibroblasts
PBMCs: peripheral blood mononuclear cells
PBS: phosphate-buffered saline
PD: Parkinson's disease
PMSF: phenylmethane sulfonyl fluoride
