# Supplementary material for: The Parkinson's disease VPS35[D620N] mutation enhances LRRK2-mediated Rab protein phosphorylation in mouse and human
Source: Biochem J. 2018 Jun 6;475(11):1861–83. doi: 10.1042/BCJ20180248 (PMC5989534; doi:10.1042/BCJ20180248)
Supplement: Supplementary Figures and Table [file BCJ-475-1861-s1.pdf]

**Supplementary Figure 1. Characterisation of the rabbit monoclonal Rab8A-total (MJF-22-74-3) antibody.** (A & B) Wild-type MEFs treated ± 100 nM MLi-2 inhibitor for 60 min prior to cell lysis. For immunoprecipitation analysis 100 µg of whole cell extract was subjected to immunoprecipitation employing either the Rab8A-total (MJF-22-74-3) antibody or Pre-immune IgG (used as a control). The immunoprecipitates were subjected to Rab8A-total (10% of sample) and Rab8-pT72 immunoblot (90% of sample) (all at 1 µg/ml primary antibody concentration). 10 µg of whole cell extract or supernatants after immunoprecipitation were subjected to Rab8A-total immunoblot. (C) The indicated lysates of A549 wild-type and A549 Rab8A knock-out cells (10 µg), HEK293 cells (10 µg), human PBMCs (5 µg), wild-type MEFs (10 µg), as well as mouse lung, spleen, kidney and brain lysates (20 µg) were subjected to Rab8A-total (MJF-22-74-3) immunoblot analysis.

**Supplementary Figure 2. Characterisation of the rabbit monoclonal Rab12-pS106 (MJF-25-9-1) antibody.** (A) HEK293 cells were co-transfected with plasmids encoding the LRRK2[Y1699C] pathogenic mutation and the indicated Rab protein that all possessed an N-terminal HA tag. 36 hours post transfection, cells were treated ± 150 nM MLi-2 inhibitor for 90 min prior to cell lysis. 0.5 µg of whole cell extract was subjected to quantitative immunoblot analysis with the indicated antibodies (all at 1 µg/ml primary antibody concentration), and the membranes developed using the Odyssey CLx scan Western Blot imaging system. (B) The indicated wild type and Rab12 knock-out A549 cells were treated ± 100 nM MLi-2 for 90 min prior to cell lysis. Generation of the Rab12 knock-out cells is described in the materials and methods. 30 µg of whole cell extract was subjected to quantitative immunoblot analysis with the indicated antibodies (all at 1 µg/ml primary antibody concentration). The membranes developed using the Odyssey CLx scan Western Blot imaging system. A duplicate Rab12-pS106 immunoblot analysis was developed using enhanced chemiluminescence (ECL) for comparison.

**Supplementary Figure 3: Three structurally unrelated LRRK2 inhibitors reduce elevated Rab10 phosphorylation in dose dependent manner in homozygous VPS35[D260N] knock-in cells.** Homozygous VPS35[D620N] knock-in and wild-type MEFs were treated with the indicated doses of MLi-2 [34, 35], GSK2578215A [36] and PF-06447475 [37] LRRK2 inhibitor for 60 min prior to cell lysis. 7.5 µg of whole cell extracts were subjected to quantitative immunoblot analysis with the indicated antibodies (all at 1 µg/ml primary antibody concentration), and the membranes developed using the Odyssey CLx scan Western Blot imaging system. Immunoblots were quantified by LiCor and presented as average ± SEMπ. Each lane represents cell extract obtained from a different dish of cells.

**Supplementary Figure 4: Flow cytometry analysis to determine the purity and viability of isolated monocytes and neutrophils, and the composition of PBMC.**
Purified monocytes were stained with the monocyte specific marker CD14 employing anti-human CD14 V500 antibody while purified neutrophils were stained with the granulocyte specific marker CD66b using anti-human CD66b FITC antibody. Cells were also stained with the cell viability dye DAPI. Cells were analysed by flow cytometry revealing that isolated monocytes had a viability >99% and a purity 87-90%, while isolated neutrophils had a viability >99% and a purity 94-98%. PBMC were stained with the B cell marker CD19 APC, the T cell marker CD3 PE, the monocyte marker CD14 V500, the granulocyte marker CD66b FITC and

DAPI. * corresponds to the monocyte and granulocyte parent gate. The proportion of each cell type within the isolated PBMC is provided. The data shown is for Donor A. A summary of the viability and purity data for all three donors is provided in Figure 6A.

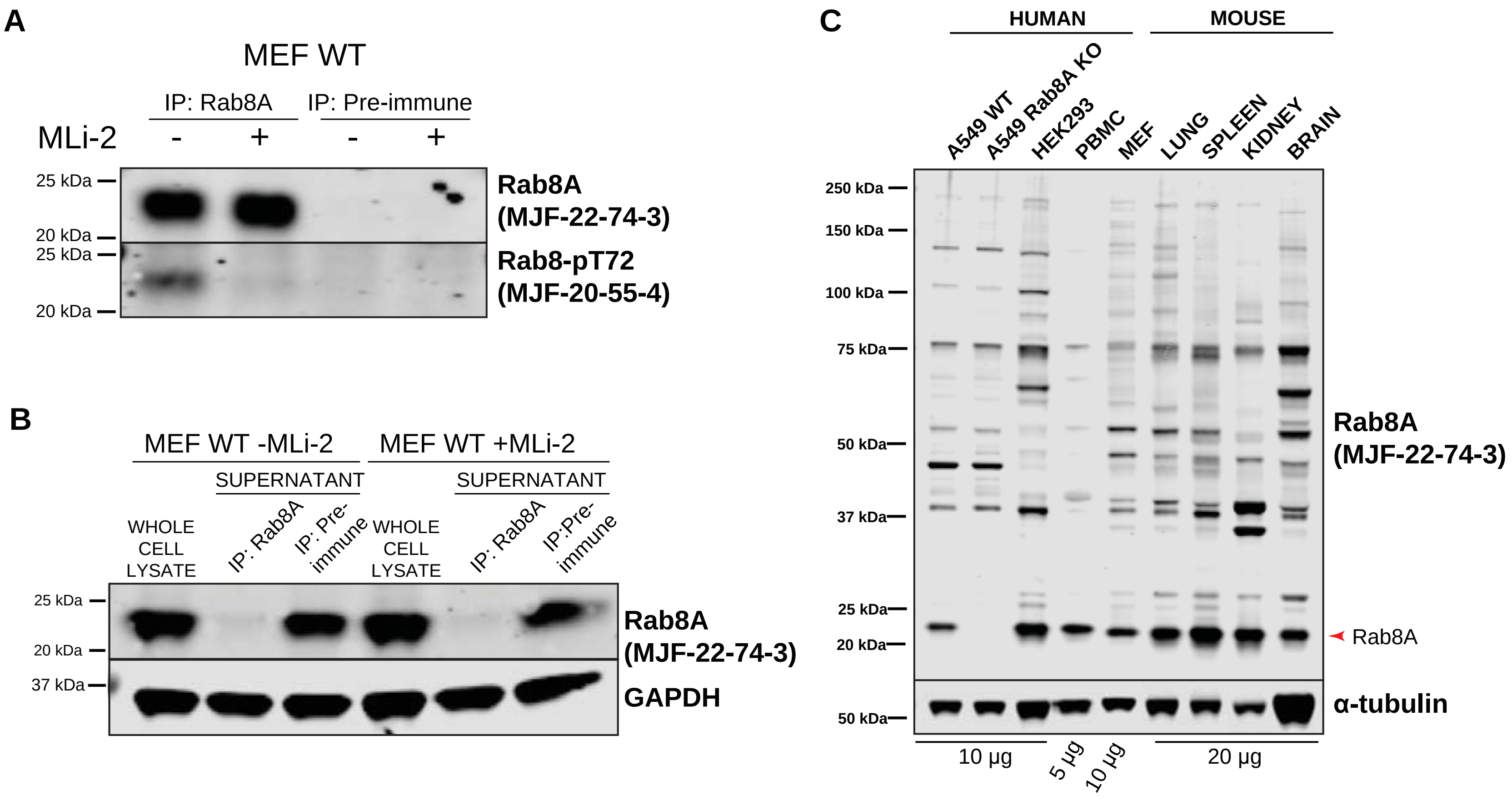


**Supplementary Figure 1**

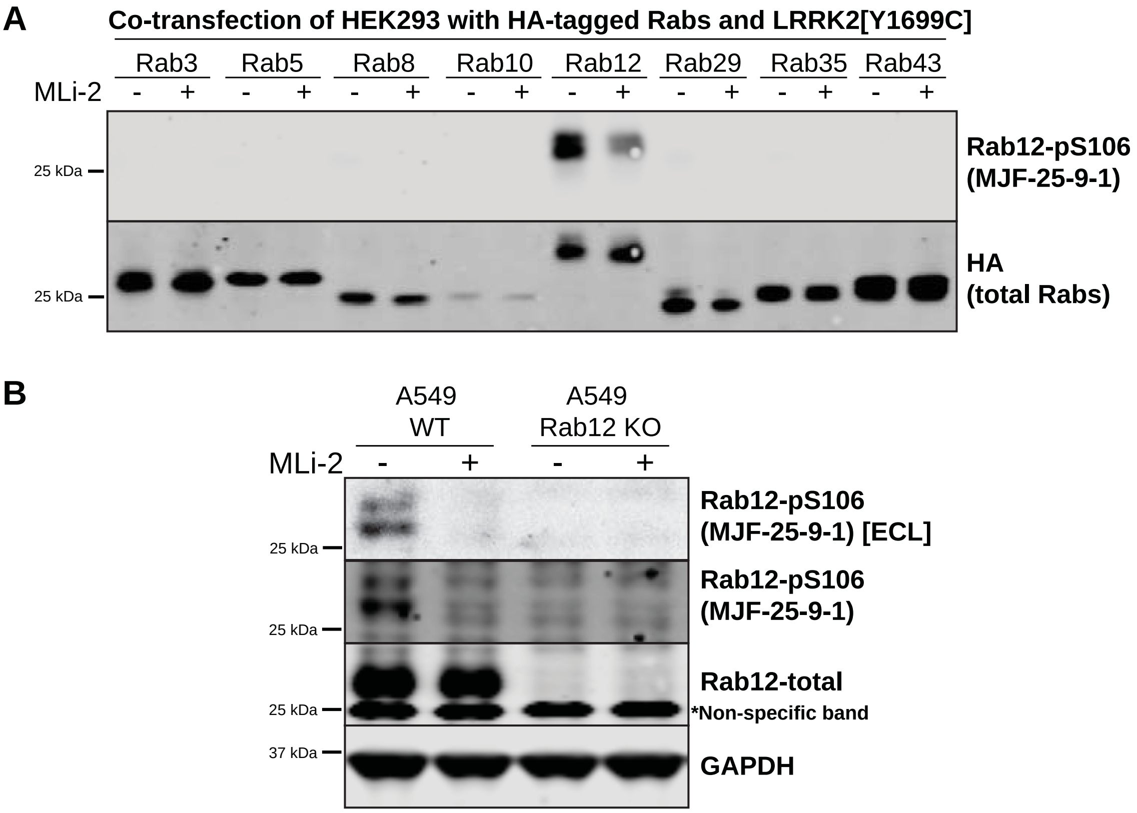


**Supplementary Figure 2**

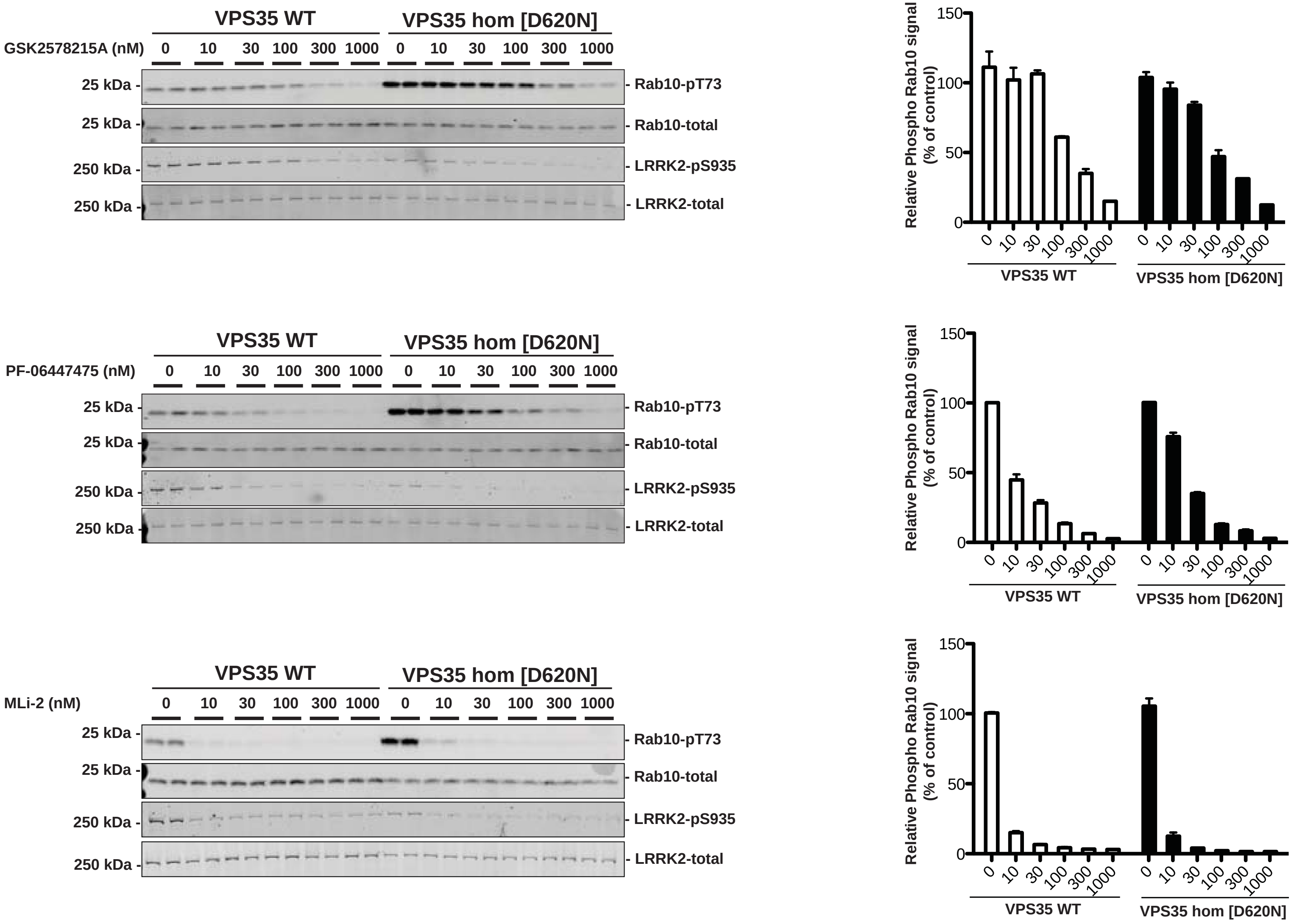


# Supplementary Figure 3

# S Figure 4

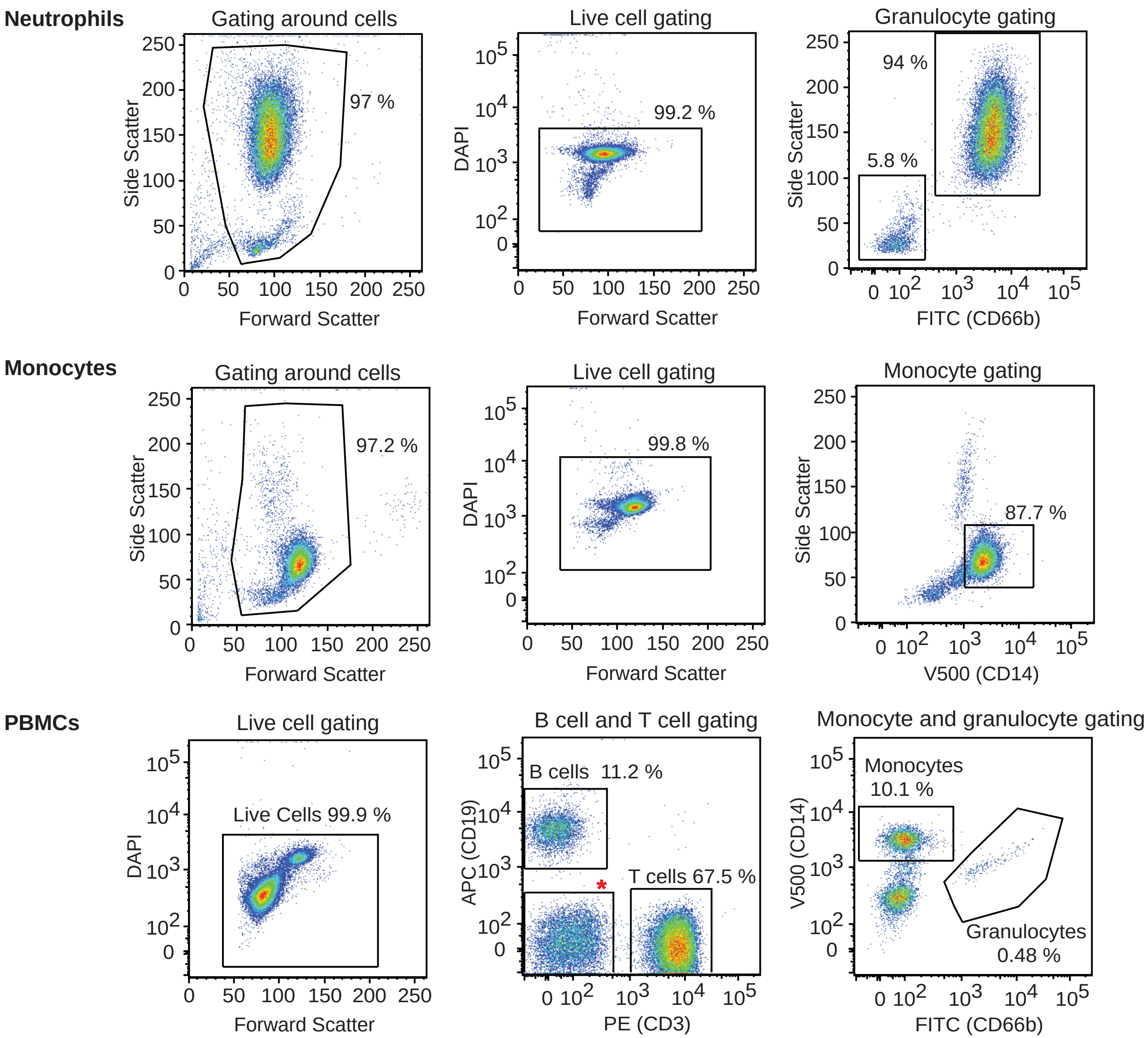

Neutrophils
Gating around cells
Side Scatter
Forward Scatter
97 %
Live cell gating
DAPI
99.2 %
Granulocyte gating
94 %
5.8 %
FITC (CD66b)
Monocytes
Gating around cells
97.2 %
Live cell gating
99.8 %
Monocyte gating
87.7 %
V500 (CD14)
PBMCs
Live cell gating
Live Cells 99.9 %
B cell and T cell gating
B cells 11.2 %
T cells 67.5 %
APC (CD19)
PE (CD3)
Monocyte and granulocyte gating
Monocytes
10.1 %
Granulocytes
0.48 %
V500 (CD14)
FITC (CD66b)

**Supplementary Table S1: Demographic and clinical data for subjects analysed in Figures 4 and 6. Abbreviations iPD idiopathic Parkinson's disease; N/A not applicable; Age at Onset of Parkinson's disease AAO**

| Donor number | Group | Gender | Age | AAO |
|---|---|---|---|---|
| 1 | iPD | m | 57 | 50 |
| 2 | iPD | f | 66 | 52 |
| 3 | iPD | m | 71 | 69 |
| 5 | iPD | f | 53 | 50 |
| 6 | iPD | m | 75 | 68 |
| 7 | iPD | m | 82 | 72 |
| 8 | iPD | m | 58 | 55 |
| 9 | Control non-PD | f | 42 | N/A |
| 12 | iPD | m | 80 | 75 |
| 13 | iPD | m | 65 | 60 |
| 14 | Control non-PD | f | 27 | N/A |
| 15 | VPS35het D620N | f | 64 | 51 |
| 16 | Control non-PD | m | 65 | N/A |
| 17 | VPS35het D620N | f | 75 | 47 |
| 18 | Control non-PD | m | 58 | N/A |
| 19 | Control non-PD | m | 44 | N/A |
| 22 | Control non-PD | m | 52 | N/A |
| 24 | Control non-PD | m | 60 | N/A |
| 25 | VPS35het D620N | f | 54 | 47 |
| 26 | Control non-PD | f | 56 | N/A |
| 27 | Control non-PD | f | 70 | N/A |
